# A Novel Functional Ingredient Derived From a Mixture of Mulberry (*Morus alba* L.) Leaves and Butterfly Pea (*Clitoria ternatea* L.) Flowers Enhances Rapid Eye Movement Sleep, Cognitive Function, and Anxiolytic Behavior via GABA_A_ Receptor‐Dependent Mechanism in Rats

**DOI:** 10.1155/omcl/2305848

**Published:** 2026-02-14

**Authors:** Jakkrit Nukitram, Aonvara Kanjanavattana, Panlekha Rungruang, Nattaporn Yotyatthai, Pannita Kaewudom, Pichayapa Promkasikorn, Patharakan Kaowsuwan, Nobuhiro Zaima, Dania Cheaha, Wipawee Thukhammee, Jintanaporn Wattanathorn

**Affiliations:** ^1^ Department of Biology, Faculty of Science, Khon Kaen University, Khon Kaen, 40002, Thailand, kku.ac.th; ^2^ Electrophysiology Laboratory (ElephLab), Khon Kaen University, Khon Kaen, 40002, Thailand, kku.ac.th; ^3^ Human High Performance and Health Promotion (HHP&HP) Research Institute, Khon Kaen University, Khon Kaen, 40002, Thailand, kku.ac.th; ^4^ Molecular Medicine Program, Multidisciplinary Unit, Faculty of Science, Mahidol University, Bangkok, 10400, Thailand, mahidol.ac.th; ^5^ Department of Medical Sciences, Regional Medical Sciences Center 5 Samut Songkhram, Ministry of Public Health, Samut Songkhram, 75000, Thailand, moph.go.th; ^6^ Department of Applied Biological Chemistry, Graduate School of Agriculture, Kindai University, 204–3327 Nakamachi, Nara City, Nara, 631–8505, Japan, kindai.ac.jp; ^7^ Biology Program, Division of Biological Science, Faculty of Science, Prince of Songkla University, Hatyai Campus, Songkhla, 90110, Thailand, psu.ac.th; ^8^ Department of Physiology, Faculty of Medicine, Khon Kaen University, Khon Kaen, 40002, Thailand, kku.ac.th

**Keywords:** anxiolytic behavior, butterfly pea flowers, cognitive function, GABA_A_ receptors, mulberry leaves, REM sleep

## Abstract

The neuropharmacological benefits for sleep quality and mental health from the extracts of *Morus alba* L. leaves (MA) and *Clitoria ternatea* L. flowers (CT) have been revealed previously. However, due to synergistic interactions of polyherbal ingredients, the positive effects of MA mixed with CT are still controversial. Preliminary outcomes from in vitro assessment exposed that a 3:1 ratio of MA:CT (MACT) yielded the highest antioxidant capacity and gamma‐aminobutyric acid (GABA)‐promoting activity among seven combination ratios: 0MA:1CT, 1MA:0CT, 1MA:1CT, 1MA:2CT, 1MA:3CT, 2MA:1CT, and 3MA:1CT. Male Wistar rats (*n* = 6/group) were electroencephalographically and electromyographically monitored to confirm the sedative‐hypnotic function of the assigned ingredients over 3 h after oral administration. Cognitive and anxiolytic effects were also evaluated thereafter. Following drug administration, it was found that MACT exhibited positive influences in a dose‐dependent manner (125, 250, and 500 mg/kg), and in a significantly better manner than either 500 mg/kg MA or CT alone. Interestingly, a majority of these effects, including sedative‐hypnotic parameters, that is, decreasing of latency to rapid eye movements (REMs) sleep and wake duration, increasing of REM sleep duration, number of REM sleep bouts, as well as elevating of cognitive function and anxiolytic parameters was reversed by pretreatment with bicuculline methiodide (2 mg/kg), a GABA_A_ receptor antagonist. Overall, the advantages of MACT‐based polyherbal drugs, which act preferentially on GABA_A_ receptors, may pave the way for further development of MACT as an alternative drug or food supplement for neuropharmacological improvements in GABA_A_ receptor‐related disorders.

## 1. Introduction

Insomnia or inadequate sleep is a health issue that has been extensively documented to have deleterious impacts globally on approximately 16.2% [1]. Insomnia and poor sleep quality dramatically decline memory and cognitive performance, as well as enhance the risk of brain degeneration and mortality [2]. In addition, sleep problems have been concomitantly seen with anxiety as they share a common mechanism, mediating through gamma‐aminobutyric acid (GABA)ergic downregulation and oxidative stress generation [3, 4]. To overcome these problems, meditation, exercise, and taking standard drugs have been provided as a variety of alternative treatments [5–7]. So far, the collected data have strongly suggested that drug administration, such as benzodiazepines (BZs), has garnered the most attention due to its ability to produce quick effects initially, followed by a sustained response through allosteric modulation on the BZ site of the GABA_A_ receptor. This eventually led to neuronal hyperpolarization mediated by Cl^−^ influx [8]. However, due to the specific site of this drug interaction, patients experiencing drug tolerance and dependence have typically been found to be a big issue with this kind of chronic treatment [9]. Therefore, there is an urgent need to develop pharmacological products from alternative sources, particularly natural ones, as they are believed to produce comparable effects to standard agents and effectively alleviate abnormal symptoms, as well as minimize harmful consequences. In addition, much better effects of drugs derived from natural sources have consistently been documented in the form of polyherbal formulations through interaction on multiple targets simultaneously to counteract some undesirable outcome [10], enhance stability or bioavailability, and decrease the required therapeutic dose of drugs [11, 12].

Mulberry leaves (*Morus alba* L.; MA) are widely utilized in northeastern Thailand as they serve as an upstream material for weaving, which is a main social activity of people in this region. In addition to GABA, several other chemical compounds have been chemically identified and found in high concentrations in MA, such as rutin, quercetin (QUE), and kaempferol (KAF) [13]. Previously, researchers evidenced that rutin and MA were found to be potent antioxidants and GABA enhancers via suppressing the activity of GABA‐metabolizing enzymes, for example, GABA transaminase (GABA‐T) [14, 15]. These would contribute to elevated sleep quality as well as cognitive functions [16, 17]. The anxiolytic‐like effect of QUE, with mechanisms similar to those of BZs, was also explored through both in vivo and in silico assessments [18]. Moreover, QUE and KAE characterized from the MA leaf extract were proposed to be the key contributors to regulate via GABAergic and 5‐HT receptors for sleep‐improving functions [19].

Butterfly pea flowers (*Clitoria ternatea* L.; CT) are rich in various anthocyanins (ATCs), including the derivatives of delphinidin, cyanidin, and malvidin [20, 21]. These compounds were documented to modulate many compartments of the CNS functions [22, 23]. In particular, their distinctive roles in elevating GABAergic transmission by allosteric interaction with many recognition sites on the GABA_A_ receptor were also apparently informed [24]. As many targets as they interacted with, it thus allowed CT extracts to pose many neuropharmacological effects, such as enhancing learning and memory, and acting as antidepressants, anti‐stress, and anxiolytic agents [25]. In addition, CT tea or extract has been traditionally used to improve human sleep quality and anxiogenic status [26, 27].

As the details mentioned the problems that are frequently encountered during standard drug use, as well as the possible neuropharmacological benefits through synergistic interaction between MA and CT. So far, no information is available on this herbal mixture from this perspective. Thus, this study aimed to evaluate the beneficial effects of MA combined with CT in an appropriate ratio compared to those of individual plant extracts through in vitro and in vivo assessments on demonstrating sedative‐hypnotic, cognitive, and anxiolytic effects. As neuronal hyperpolarization, induced by the GABA_A_ receptor, can be accomplished through multisite interaction on its pentameric subunits [8], and chronic activation of the allosteric BZ site by BZs, but not direct activation of the GABA_A_ receptor, has been proposed to be more susceptible to drug tolerance [28]. Hence, the possible underlying mechanism was investigated with an emphasis on the modulation of the GABA_A_ receptor active site.

## 2. Materials and Methods

### 2.1. Preparation of Plant Materials

All plant materials used in this study were harvested from Nonruang village No. 6, Baankho Sub‐district, Muang Khon Kaen District, Khon Kaen Province, Thailand, between September and October 2023. After that, a taxonomist, Dr. Pornchai Kladwong, verified the authenticity of both MA (Panya, T. 6 KKU No. 25976) and CT (Panya, T. 4 KKU No. 25544) at the KKU herbarium, Khon Kaen University, where the voucher specimens have been kept. Leaves of MA and flowers of CT were used in this study. The MA extraction process followed the method outlined in previous research [15, 29]. Initially, MA was cleaned and then used to prepare an aqueous decoction at 60°C for 20 min (2.5% w/v), followed by filtration through nylon cloth to obtain the supernatant. For the CT extract, the cleaned flowers were macerated in room temperature water for 3 days (5% w/v), with the mixture stirred every 24 h to ensure thorough mixing. The extract was then filtered using Whatman No. 1 filter paper, and the pH of the supernatant was adjusted to 4.3. Both extracts were then combined with 5% maltodextrin, an encapsulation matrix, and dried using a Mini Spray Dryer B‐290 (Buchi Co. Ltd., USA) to produce extracted powder [29, 30]. The production yields of the MA and CT extracts were 43.33% w/w and 54.28% w/w of the fresh sample, respectively. Both extracts were stored in dark bottles at −20°C until used.

Different combination ratios of MA and CT, that is, 0MA:1CT, 1MA:0CT, 1MA:1CT, 1MA:2CT, 1MA:3CT, 2MA:1CT, and 3MA:1CT, were tested to assess preliminary outcomes through an in vitro experiment, as detailed in this study. Among them, it was found that a ratio of 3:1 of MA:CT, abbreviated as MACT, demonstrated the highest antioxidant capacity and GABA‐promoting activity. Therefore, MACT was subjected to confirm a more precise main bioactive compound and its positive effects through the high‐performance liquid chromatography (HPLC) method and in vivo assessment, respectively, compared to MA and CT alone.

### 2.2. In Vitro Evaluation of Major Bioactive Compounds

#### 2.2.1. Total Phenolic Contents (TPCs)

The TPCs were measured using the Folin–Ciocalteu method in 96‐well microtiter plates. Fresh reagents were prepared, consisting of 25 µL of Folin–Ciocalteu reagent (50% v/v) (Sigma–Aldrich, USA), 50 µL of the plant extract, and 125 µL of Na_2_CO_3_ (20% w/v) (Sigma–Aldrich, USA). The mixture was incubated at room temperature (30 ± 2°C) in the dark for 40 min. Absorbance was then measured using a microplate reader (iMark Microplate Absorbance Reader) at 700 nm. Gallic acid (GAL) (15.625–250 µg/mL) was used as a standard to generate a calibration curve for calculating the TPCs. The data were expressed as mg GAL equivalent per gram of extract. The assay was performed in triplicate [15].

#### 2.2.2. Total Flavonoid Contents (TFCs)

The aluminum chloride colorimetric method quantified the TFCs. Briefly, either QUE (6.25–100 µg/mL) as a standard or 50 µL of the plant extract was combined with 200 µL of aluminum chloride (5% w/v). The reaction occurred for 30 min at room temperature (30 ± 2°C). Absorbance was then measured with a microplate reader at 437 nm to create the calibration curve [31]. The results were reported as mg QUE equivalent per gram of extract. The data were presented as the average of three replicates.

#### 2.2.3. Total ATC Contents (TACs)

The TACs were spectrophotometrically measured using the pH differential method, which relies on ATCs’ structural transformation and color changes due to pH variations [32]. 1 mL of plant extract was mixed with 2 mL of a pH buffer—potassium chloride (0.025 M) for pH 1.0 or sodium acetate (0.4 M) for pH 4.5. Absorbance was recorded using a UV‐spectrophotometer (Pharmacia LKB‐Biochrom 4060) at 520 and 720 nm after the mixture was incubated at room temperature (30 ± 2°C) for 10 min. The experiments were performed in triplicate, and the results were expressed as cyanidin‐3‐glucoside (C3G) equivalents/L, calculated using the following equation:

Absorbance A=A520–A700 at pH 1.04.5 – A520–A700 at pH ,

TACs mg/L=A×MW×DF× 103/ε×1,

where MW = molecular weight of C3G at 449.2 g/mol; DF = dilution factor obtained from the experiment; *ɛ* = molar extinction coefficient at 26,900 in L/mol/cm, for C3G; 10^3^ = conversion factor changed from g to mg; 1 = path length of the cuvette (1 cm).

### 2.3. In Vitro Evaluation of Biological Activities

#### 2.3.1. The 1,1‐Diphenyl‐2‐Picrylhydrazyl (DPPH) Radical Scavenging Assay

Following the recent protocol [15], plant extract (5–1000 µg/mL) at 20 µL was mixed with 180 µL of DPPH in methanol (0.15 mM). The reaction was incubated for 30 min in the dark at room temperature (30 ± 2°C). The free radical scavenging ability was evaluated by observing the decolorization of DPPH●, as it received protons from the plant extract, at an absorbance of 517 nm using a microplate reader (iMark Microplate Absorbance Reader). Trolox (µg/mL) (Sigma–Aldrich, Germany) was used as a positive control. The percentage of DPPH radical scavenging was calculated using the following equation:

Percentage of DPPH inhibition=1−A/B×100,

where *A* is the absorbance in the presence of plant extract; *B* is the absorbance in the absence of plant extract.

The percentage of DPPH inhibition was plotted against the concentration of different plant extracts to derive the linear equation, which was then used to determine the concentration (mg/mL) needed to inhibit 50% of DPPH radical formation (IC_50_).

#### 2.3.2. The Ferric‐Reducing Antioxidant Power (FRAP) Assay

Following the methodology from a previous study [11], we used the FRAP assay to evaluate the antioxidant capacity of the plant extract. The experiment began by mixing 1.45 mL of FRAP reagent, which contained a 10:1:1 ratio of solution A (300 mM acetate buffer, pH 3.6), solution B (10 mM 2,4,6‐TPTZ in 40 mM HCl), and solution C (20 mM ferric chloride), with 50 µL of the plant extract. The reaction was then incubated in a water bath at 37°C for 10 min. As the plant extract reduced the ferric tripyridyltriazine (Fe^3+^ TPTZ) complex to its ferrous form, a blue color developed, which was recorded using a spectrophotometer (Pharmacia LKB‐Biochrom4060) at 593 nm. The data were analyzed and expressed as IC_50_, with Trolox used as the standard. All tests were performed in triplicate.

#### 2.3.3. GABA‐T Suppressive Effects

We followed the procedure outlined in a previous study [15] to assess the potential effects of plant extract on GABA‐T inhibitory activity. In brief, the rat cerebral cortex was isolated and homogenized in 0.1 M potassium phosphate buffer, pH 7.4 (1:5 w/v). The supernatant, which served as the source of GABA‐T, was collected after cooling and centrifugation (4°C) at 12,000 rpm for 10 min. An assay solution was then prepared by mixing 200 µL of the plant extract, 200 µL of brain supernatant, and 800 µL of GABA‐T buffer (containing 20 mM GABA, 10 mM *α*‐ketoglutarate, and 0.5 mM NAD in 0.5 M sodium phosphate buffer, pH 8.0), and incubated at 21°C for 30 min. GABA‐T inhibition was monitored spectrophotometrically by measuring the production of NADH from NAD^+^ at 340 nm. Each sample was tested in triplicate, and the results were analyzed and expressed as IC_50_.

### 2.4. The HPLC Fingerprint of Plant Extracts

The HPLC method was adapted from previous studies [33, 34]. The system consisted of 515 HPLC pump and 2998 photodiode array detectors (Water Company, USA). Chromatographic separation was performed using a column Poroshell 120 EC‐C18 column (Agilent Technologies, USA) with a guard column Poroshell 120 EC‐C18 column (5 × 4.6 mm id, 4.0 µm) (Agilent Technologies, USA). Acetonitrile (A), 0.1% formic acid (B) were used as mobile phases for quantitation of phenolic and flavonoid compounds by setting the gradient elution at a flow rate of 1.0 mL/min with the following gradient: 0 min, 90% B; 10 min, 75% B; 20 min, 40% B; 30 min, 30% B and 35 min, 20% B. Whereas, the quantitation of ATCs used the mobile phases as 10% formic acid in deionized water (10:90 v/v) (A), acetonitrile (B) by setting the gradient elution at a flow rate of 1.0 mL/min with the following gradient: 0 min, 93% A; 35 min, 73% A; 45 min, 35% A; 47 min, 30% A; 50 min, 20% A; 55 min, 20% A; 60 min, 10% A. The sample was filtered (0.22 μm, CNW, China), and a direct injection of the tested sample at the volume of 20 mL on the column was carried out. The chromatograms were recorded at 254, 275, 280, 310, 320, and 370 nm (for phenolics and flavonoids detection), and 530 nm (for ATCs detection) using a UV detector and data analysis was carried out using EmpowerTM 3 software.

### 2.5. Animals Preparation and Experimental Protocol

The experimental protocols for this study received approval from the Institutional Animal Care and Use Committee (IACUC) at Khon Kaen University, following the Ethics of Animal Experimentation established by the National Research Council of Thailand, as well as the guidelines set forth by the European Science Foundation (Use of Animals in Research, 2001), the Animal Research: Reporting of In Vivo Experiments (ARRIVE), and the International Committee on Laboratory Animal Science (ICLAS, 2004). The project number was recorded as IACUC‐KKU‐140/66, with reference No. 660201.2.11/744 (152), and was approved on December 21^st^, 2023. Adult male Wistar rats were bred and housed at the Northeast Laboratory Animal Center of Khon Kaen University, in a controlled environment with temperatures maintained between 23–25°C, 50%–55% relative humidity, and a 12‐h light/dark cycle. The rats were given commercial pellet food and distilled water *ad libitum*. The experiment began once the animals reached the appropriate age (3 months after birth and weighing between 250 and 300 g) to prevent skull fragility during surgery.

### 2.6. Implanting the Electroencephalogram (EEG) and Electromyogram (EMG) Electrodes

To accurately quantify and monitor sleep microarchitecture in rodents, thus the electrodes of the EEG and EMG were implanted. The method for electrode implantation required to obtain these biological signals was adapted from recent publications [35, 36]. Briefly, rats were subcutaneously administered 0.05 mg/kg atropine sulfate (T.P. Drug Laboratories, 1969, Co., Ltd., Thailand) for 15 min to reduce the secretion of saliva and fluid in the respiratory tract. Following this, a mixed solution of 10 mg/kg xylazine (L.B.S. Laboratories, Co., Ltd., Thailand) and 60 mg/kg zoletil (Virbac, Co. Ltd., Thailand) was intraperitoneally injected to induce deep anesthesia, which was assessed by the pinch withdrawal reflex in the hind legs. After securing the head with a stereotaxic apparatus, the scalp was injected with the local analgesic lidocaine (Locana, L.B.S. Laboratories, Co., Ltd., Thailand) and subsequently exposed through a midline incision. The coordinates defined from the bregma in the anteroposterior (AP) and mediolateral (ML) axes were specified according to the rat brain atlas [37] for the left parietal cortex (PC) (AP: −4 mm, ML: 4 mm) and cerebellum (AP: −11.8 mm, ML: 0 mm) for recording and referencing purposes, respectively. The skulls in the designated areas were drilled to accommodate silver wire electrodes (A–M System). In addition, two electrodes for capturing the EMG were inserted into the neck muscles for bipolar recording. Finally, extra holes were made for tightening stainless screws, and dental cement (General Drug House Co., Ltd., Thailand) was applied to secure the electrodes firmly to the skull. During the post‐surgery period, all animals received intramuscular injections of ampicillin (100 mg/kg) (General Drug House Co., Ltd., Thailand) and carprofen (10 mg/kg) (Zoetis, Co., Ltd., Thailand) to prevent infection and alleviate pain, respectively. Yunnan Paiyao (Yunnan Baiyao Group Co., Ltd., China) was also applied to stop bleeding during and after the surgical procedures.

### 2.7. Experimental Design

In this study, we divided the animals into two experimental sets.

#### 2.7.1. Experiment 1

Sedative‐hypnotic, cognitive, and anxiolytic effects of the MACT in a dose‐dependent manner and in comparison to either MA or CT *per se*.

To initiate the experiment, all rats were acclimated to the experimental apparatus and environments for 3 days. The following day, the rats were divided into six groups (*n* = 6) to receive oral administration of 5% maltodextrin solution (Control), 125 mg/kg MACT (MACT125), 250 mg/kg MACT (MACT250), 500 mg/kg MACT (MACT500), 500 mg/kg MA (MA500), and 500 mg/kg CT (CT500).

#### 2.7.2. Experiment 2

Sedative‐hypnotic, cognitive, and anxiolytic effects of MACT in GABA_A_ receptor‐dependent conditions.

This experiment was designed to determine the potential underlying mechanisms of MACT in mediating sedative‐hypnotic, cognitive, and anxiolytic effects. After 3 days of habituation, animals were divided into four groups (*n* = 6) to receive 5% maltodextrin solution (Control), 500 mg/kg MACT (MACT500), 2 mg/kg bicuculline methiodide (Bi) (Sigma–Aldrich, St. Louis, MO, USA, #102674474), a competitive antagonist of the GABA_A_ receptor [3], and 2 mg/kg Bi + 500 mg/kg MACT (BiMACT500). MACT was administered orally immediately after Bi was given intraperitoneally. To minimize animal use in accordance with the 3R principles, the rats in the first two groups of this experiment were sourced from Experiment 1.

### 2.8. Experimental Setup

**Figure 1:**
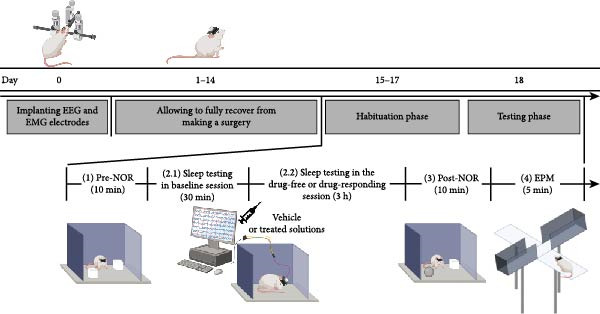
Schematic diagram of the experimental setup (the BioRender.com platform was used to create this figure).

The experimental setup for data collection was adapted from previous studies [35, 36]. In brief, after allowing the animals to fully recover from surgery for at least 2 weeks (days 0–14), they underwent a habituation phase lasting three consecutive days (days 15–17). Each day of this phase included the following sequence of behavioral assessments and data acquisition: (1) pretreatment period of the novel object recognition (NOR) test (pre‐NOR) (10 min), (2) sedative‐hypnotic test (3.5 h, with the first 30 min serving as the baseline session [2.1] and the remaining 3 h as the drug‐free session [2.2]), (3) posttreatment period of the NOR test (post‐NOR) (10 min), and (4) elevated plus‐maze (EPM) test (5 min). In the testing phase (day 18), the procedure was similar to that of the habituation phase, except that drug administration occurred immediately before the final 3 h of the second step (Figure 1).

### 2.9. Data Acquisition

#### 2.9.1. Electrophysiological Signals and Sedative‐Hypnotic Test

The setting of instrumental devices for data recording followed our recent publications [38, 39]. The analog data of biological signals were transmitted through the PowerLab 26T (AD Instruments, Castle Hill, NSW, Australia) for amplification and digitization, respectively. To prevent signal distortion known as aliasing due to the 0.5–100 Hz band‐pass digital filter, data were collected at a sampling rate of 2 kHz, following the Nyquist rate to exceed the highest frequency adequately. Power line artifacts were removed at 50 Hz. Raw data were collected with LabChart 7.3.7 Pro software for offline analysis. To monitor the sleep profile and examine the sedative‐hypnotic effects of drugs, animals were connected to electrophysiological devices via an electrical line and placed in a square box (80 cm long × 80 cm wide × 50 cm high) for 3.5 h [35, 40] (step 2 in Figure 1). The first 30 min of this task were designated for the baseline session and used as an internal reference level of body function for data normalization relative to drug‐responsive effects following 3 h of drug administration. The sleep parameters included sleep latency to non‐rapid eye movement (NREM) and REM sleep, time/percentage of sleep/wake stage duration, number of bouts, and average bout durations in each stage of sleep/wake cycle [36].

#### 2.9.2. Cognitive Function Test by the NOR Task

We adapted the protocol of the NOR test from a previous study [11]. During both pre‐NOR and post‐NOR sessions (step 1 and 3 in Figure 1) of the habituation phase, animals were placed in a square box (80 cm long × 80 cm wide × 50 cm high) and allowed to explore freely for 10 min. The box contained two identical objects placed opposite each other on the floor, referred to as familiar objects. In the post‐NOR session of the testing phase, one of the familiar objects was replaced with a novel object that differed in shape, size, and texture to familiar one. This change was made to assess nonspatial memory in rats by calculating the novel object preference index (NOPI). A higher NOPI for the novel object indicates better memory and cognitive function, as rodents typically exhibit exploratory behavior toward novel stimuli. NOPI was calculated using the following equation:

NOPI=X2–Y2/X2+Y2−X1–Y1/X1+Y1×100,

where *X*_1_ = time spent at the first object in the pre‐NOR period during the testing phase; *Y*_1_ = time spent at another object in the pre‐NOR period during the testing phase; *X*_2_ = time spent at the novel object in the post‐NOR period during the testing phase; *Y*_2_ = time spent at another object in the post‐NOR period during the testing phase.

In addition, the speed and total distance of animal locomotor activity were analyzed from the NOR test to assess whether animals exhibit locomotor defects.

#### 2.9.3. Anxiety Test by the EPM Task

The EPM apparatus was positioned 40 cm above the floor. It consisted of two identical open arms (30 cm long × 5 cm wide × 0.5 cm high) and two closed arms (30 cm long × 5 cm wide × 15 cm high) arranged opposite each other, forming a 5 cm square central zone. This task is based on the principle that rodents naturally avoid exposed spaces, and the elevated structure of the maze heightens their anxiety. Therefore, an increased preference for open arms may indicate an anxiolytic effect. To initiate the experiment, rats were placed in the central zone, facing the closed arms, and were allowed to move freely for 5 min. Parameters measured during this task included the percentage of time spent and the number of entries into the open arms [12] (step 4 in Figure 1).

### 2.10. Data Analysis

#### 2.10.1. Sleep Microarchitectures

**Figure 2:**
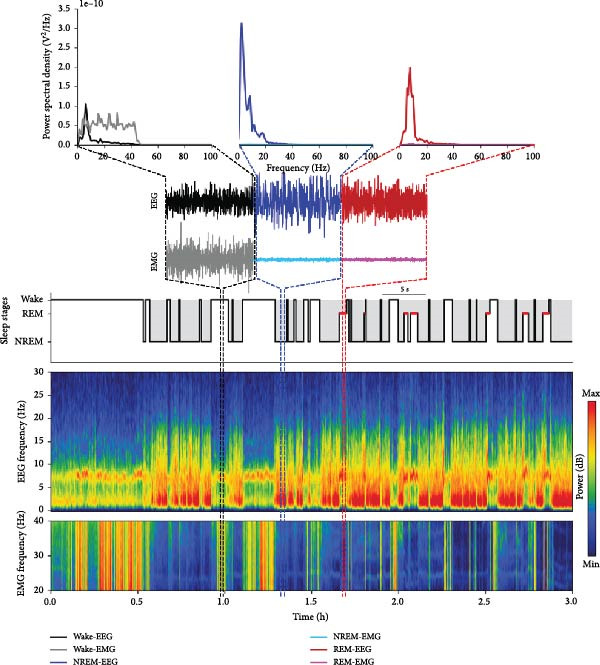
Criteria for evaluating the stages of the sleep/wake cycle.

A unique oscillation of EEG and EMG at any time point reflects each stage in sleep/wake cycle [35, 36, 40]. We, thus, developed the in‐house Python code to process and extract the meaningful parameters known as power spectral density (PSD) from these raw biological signals (EEG and EMG). The workflow diagram for analyzing sleep microarchitecture is provided (Figure S1). Briefly, the data were initially down‐sampled from 2 to 200 Hz. It was then reorganized, with the first and second channels assigned to the EEG and EMG, respectively. The preprocessed data were passed through the sliding_window function (YASA package, version 0.6.3) [41] with a 5‐s window to segment the signals for rapid analysis. These smaller segments were continuously input into the fast Fourier transform algorithm using Welch’s method (welch command of SciPy package, version 1.11.4) to compute the PSD across the delta (0.5–4.0 Hz), theta (4.0–8.0 Hz), alpha (8.0–12.0 Hz), beta (12.0–30.0 Hz), gamma I (30.0–45.0 Hz), and gamma II (60.0–95.0 Hz) frequency ranges. After finishing the data preprocessing process, the PSD in two‐data channels was automatically scored for sleep/wake stages using the AccuSleep toolbox (MATLAB version R2023a) with a setting of 5‐s epoch length and 200 Hz sampling rate [42]. Subsequently, Python code for multitaper spectrogram analysis [43] and hypnogram visualization (YASA package, version 0.6.3) [41] was applied to display changes in the spectrogram from the EEG and EMG, alongside extracting the sleep/wake stage pattern. The criteria for stage identification were in accordance with previous experiments [35, 40, 42] and detailed in the following dichotomous key (Figure 2).

1A. High power of EMG PSD caused by high muscular tone—wake.

1B. Low power of EMG PSD—2.

2A. High power of the PC PSD ranging from 0.5–20 Hz with a peak in the delta band—NREM sleep.

2B. High power of the PC PSD with a clear peak in the theta band—REM sleep.

#### 2.10.2. Animal Behaviors

During behavioral testing, we placed a webcam vertically above the animal testing apparatus to record the animal’s movement to continuously feed through the OptiMouse toolbox [44] for extracting the key parameters of each behavioral task, as previously detailed. A trained scientist was blinded in order to perform the behavioral assessments.

### 2.11. Determining the Similar/Different Degrees of Brain Modulatory Effects of Tested Substances

To evaluate the degree of feature similarity/difference from treated drugs, all significant parameters from animal behaviors and sleep/wake profiles were arranged to form a 48 × 9‐dimensional feature set (48 = the number of groups × the number of animals per group, 9 = the number of parameters). High‐dimensional data were reduced into a meaningful 2‐dimensional representation using the *t*‐Stochastic neighbor embedding (*t*‐SNE) function (scikit‐learn package, version 1.3.2) [45] for easier assessments in qualitative aspects. A 95% confidence ellipse was calculated to define the boundary for each data class. Finally, the distances between the mean centroid of each group and the projected values from each class were quantitatively measured and expressed as the maximum mean discrepancy (MMD) distance of each treatment paired [38].

### 2.12. Statistical Analyses

Data from individual animals were processed and presented as mean ± standard error of the mean (S.E.M.). A one‐way analysis of variance (ANOVA) and mixed model ANOVA were used to determine whether the variance in group means was significantly different, considering one independent variable (drug treatments) in single and repeated measurements, respectively. When applicable, Tukey’s post hoc test was conducted when the *p*‐value was less than 0.05. All statistical analyses were performed using the Python package statsmodels [46].

## 3. Results

### 3.1. In Vitro Evaluation of Major Bioactive Compounds and Biological Activities

Our preliminary study through an in vitro indicates that the MACT shows the most significant potential for enhancing GABAergic transmission and acts as a significant free radical scavenger, providing protection against oxidative stress (Table S1). Consequently, the MACT combination was selected for investigation of its neuromodulatory effects through an in vivo study and to quantify the primary bioactive compound using an HPLC method, identifying the chemical constituents that may enhance the efficacy of the combined extracts compared to the individual extracts of MA and CT.

### 3.2. The HPLC Fingerprints of Plant Extracts

The HPLC analysis identified various compounds in the phenolic and flavonoid groups, including rutin, GAL, QUE, KAF, chlorogenic acid, and ellagic acid, present in MACT, MA, and CT at different concentrations, along with several other unidentified peaks. Notably, rutin‐rich extracts of MACT were detected at 0.266 mg/g extract. To compare among three forms of plant extracts, it was found that MACT and MA were the only sources to show a significant accumulation of KAF, with the highest concentration observed in the MACT combination at 0.037 mg/g extract (Figure S2 and Table S2). For ATC quantification, cyanidin‐3‐sophoroside (C3S) and malvidin‐3‐glucoside (M3G) were the primary compounds found only in MACT and CT, and C3S was exceptionally high in the mixed extract at 0.332 mg/g extract (Figure S3 and Table S3).

### 3.3. Experiment 1

Sedative‐hypnotic, cognitive, and nootropic effects of MACT in a dose‐dependent fashion and in comparison, to either MA or CT alone.

#### 3.3.1. Sleep Microarchitectures

The sleep analysis reveals that MACT significantly exhibited sedative‐hypnotic properties in a dose‐dependent manner, as evidenced by the latency time to enter NREM and REM sleep, the time/percentage of wake, NREM, and REM durations, and the number of bouts to REM sleep, while showing no alterations in the number of bouts to wake and NREM sleep, or in the average bout durations of each sleep stage (Figure 3C,D). Therefore, MACT at a high dose (MACT500) was used for comparison with a single extract of MA and CT at the same concentration. Interestingly, although MA500 and CT500 significantly altered the positive outcomes in the aforementioned parameters, a higher therapeutic dose was required to achieve the effects of MACT500 (Figure 3A–C and Table S4).

Figure 3The sleep profile microarchitecture includes sleep latency (A), time/percentage of sleep stage duration (B), number of bouts (C), and average bout duration (D) for each stage in the sleep/wake cycle after administering MACT in a dose‐dependent manner, compared to either MA or CT alone.  ^∗^ *p* < 0.05,  ^∗∗^ *p* < 0.01,  ^∗∗∗^ *p* < 0.001,  ^∗∗∗∗^ *p* < 0.0001, based on one‐way ANOVA followed by Tukey’s post hoc test.

**Figure:**
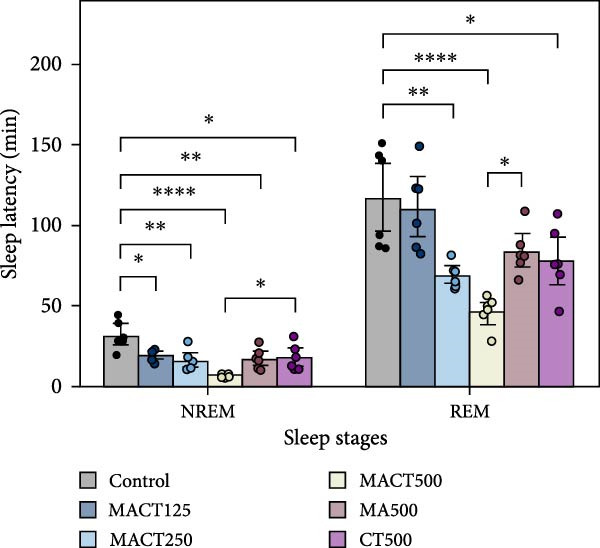
(A)

**Figure:**
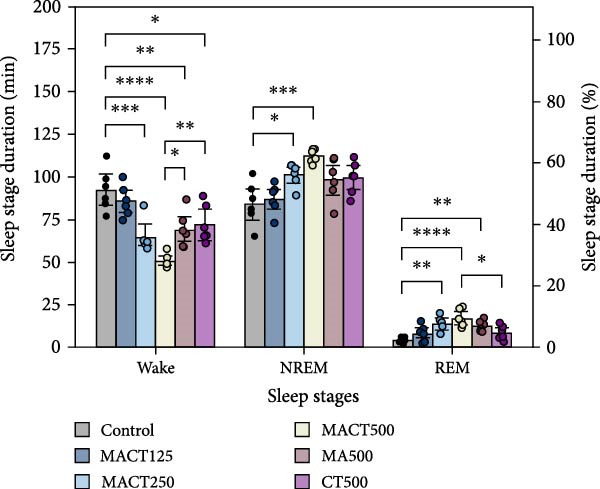
(B)

**Figure:**
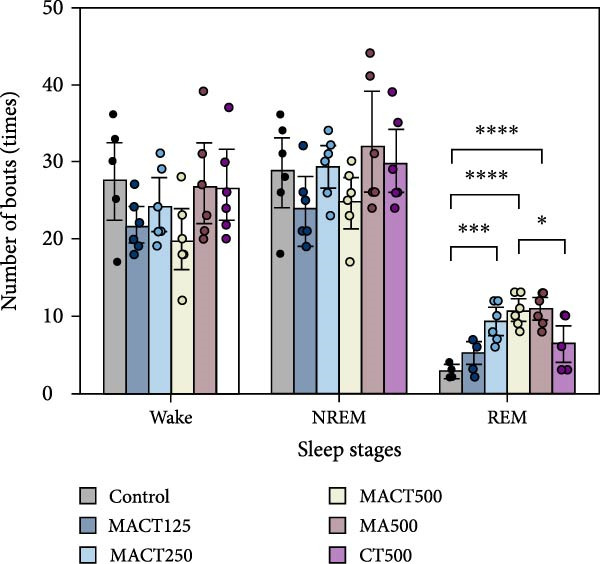
(C)

**Figure:**
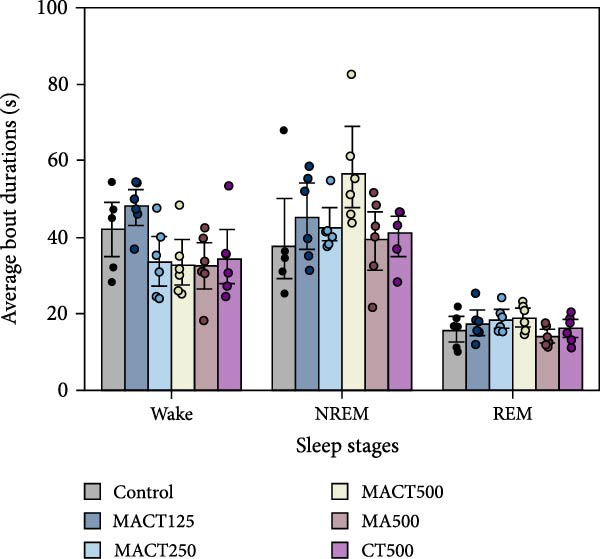
(D)

#### 3.3.2. NOR Test

We evaluated NOPI to refer to the cognitive performance and capacity of nonspatial memory in animal models. The one‐way ANOVA indicated significant changes in NOPI values (Table S4). Tukey’s post hoc analysis revealed that cognitive enhancement was clearly observed in the MACT500 (*p* < 0.0001), MA500 (*p* = 0.0018), and CT500 (*p* = 0.0186) groups; however, the rats that received CT500 exhibited a lower effect compared to those in the MACT500 group (*p* = 0.0465) (Figure 4A). Additionally, no changes were noted in the total distance moved (Figure 4B) or average speed of animal movement (Figure 4C), suggesting that these improvements are likely free from the confounding effects of impaired locomotor activity.

Figure 4Cognitive parameters obtained from novel object recognition tests include the novel object preference index (NOPI) (A), total distance moved (B), and speed of animal movement (C) following administration with MACT in a dose‐dependent manner, compared to either MA or CT alone.  ^∗^ *p* < 0.05,  ^∗∗^ *p* < 0.01,  ^∗∗∗^ *p* < 0.001,  ^∗∗∗∗^ *p* < 0.0001 under one‐way ANOVA analysis followed by Tukey’s post hoc test.

**Figure:**
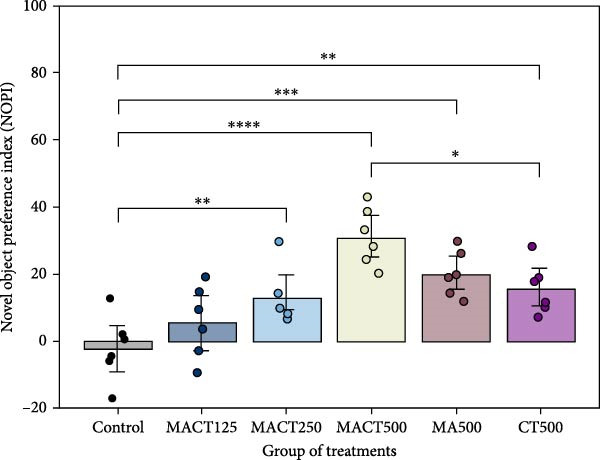
(A)

**Figure:**
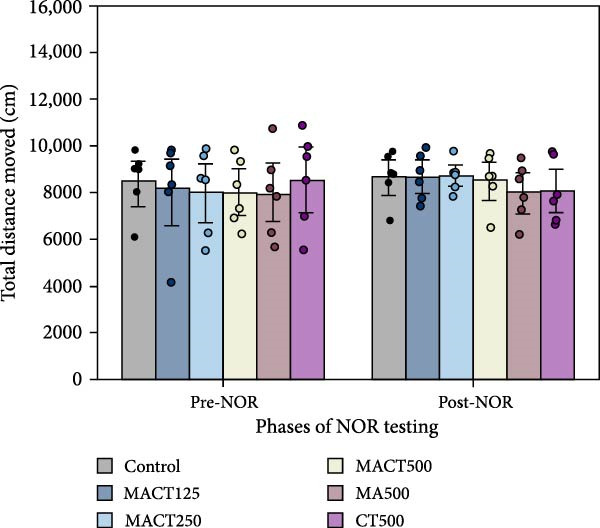
(B)

**Figure:**
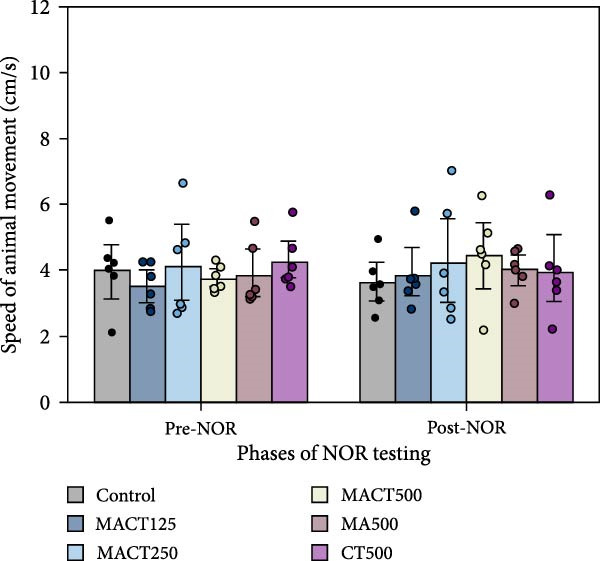
(C)

#### 3.3.3. EPM Test

Upon conducting a one‐way ANOVA analysis, it was observed that significant changes had occurred in anxiolytic parameters in rats, including the percentage of time spent and the number of entries in the open arms (Table S4). A notable increase in the first parameter was observed in rats treated with MACT500 (*p* = 0.0004), MA500 (*p* = 0.0169), and CT500 (*p* = 0.0402) (Figure 5A). Regarding the second parameter, while the number of rats administered all extracts at 500 mg/kg entering the open arms increased significantly (*p* < 0.0001, *p* = 0.0007, and *p* = 0.0062 for MACT500, MA500, and CT500 compared to the control, respectively), the increase observed in CT500‐treated rats was still significantly lower than that seen in MACT500 rats (*p* = 0.0126) (Figure 5B).

Figure 5Anxiolytic parameters obtained from elevated plus maze tests, including time spent in open arms (A) and the number of entries to open arms (B), were assessed following administration of MACT in a dose‐dependent manner, compared to either MA or CT alone.  ^∗^ *p* < 0.05,  ^∗∗^ *p* < 0.01,  ^∗∗∗^ *p* < 0.001,  ^∗∗∗∗^ *p* < 0.0001 based on one‐way ANOVA analysis followed by Tukey’s post hoc test.

**Figure:**
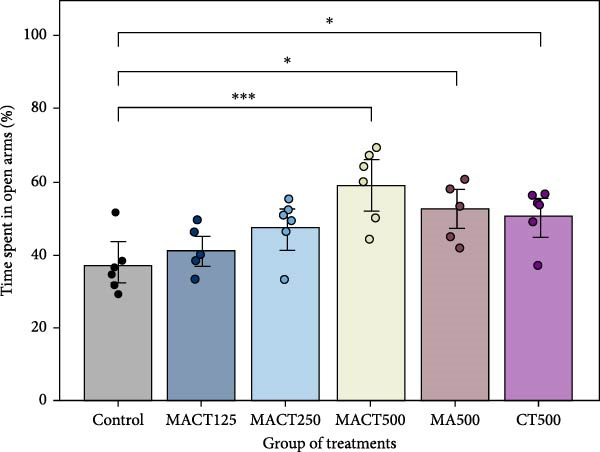
(A)

**Figure:**
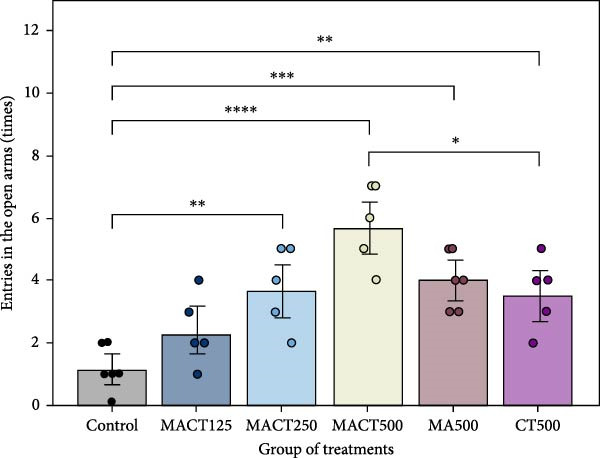
(B)

The top view of experiment 1 revealed that the treatment with MACT500 could manipulate brain functions to have greater potential than the individual extracts of MA or CT. However, the possible mechanism underlying these improvements remains to be confirmed. In this experiment, the focus was specifically on the function relying on GABA_A_ receptor activity, which was demonstrated in the subsequent experiment.

### 3.4. Experiment 2

Sedative‐hypnotic, cognitive, and anxiolytic effects of MACT in GABA_A_ receptor‐dependent condition.

#### 3.4.1. Sleep Microarchitectures

The statistical results from the one‐way ANOVA analysis demonstrated that the following parameters of sleep microarchitectures were significantly affected by MACT500 treatment, that is, latency to falling asleep in NREM and REM sleep, the time/percentage duration spent for awake, NREM, and REM sleep, and the number of bouts to REM sleep (Table S5). However, most of these parameters were abolished with Bi pre‐exposure, as observed in the BiMACT500 rats (*p* = 0.0092, *p* = 0.0233, *p* = 0.0017, and *p* = 0.0345 for latent time to REM sleep (Figure 6A), time/percentage spent awake and in REM sleep duration (Figure 6B), and the number of bouts to REM sleep (Figure 6C), respectively). Notably, no significant differences were observed in the number of bouts of wakefulness and NREM sleep or in the average bout durations for wakefulness, NREM, and REM sleep (Figure 6C,D). It is important to mention that the application of Bi alone produced no significant differences in sleep latency to NREM and REM sleep or in the time/percentage spent awake and in NREM duration while also leading to an increase in time/percentage spent in REM duration (*p* = 0.0050) (Figure 6B) and the number of bouts to REM sleep (*p* = 0.0017) (Figure 6C).

Figure 6The sleep profile microarchitecture encompasses sleep latency (A), the time/percent duration of each sleep stage (B), the number of bouts (C), and average bout duration (D) across the sleep/wake cycle after administering MACT in GABA_A_ receptor‐dependent conditions.  ^∗^ *p* < 0.05,  ^∗∗^ *p* < 0.01,  ^∗∗∗^ *p* < 0.001,  ^∗∗∗∗^ *p* < 0.0001 based on one‐way ANOVA analysis followed by Tukey’s post hoc test.

**Figure:**
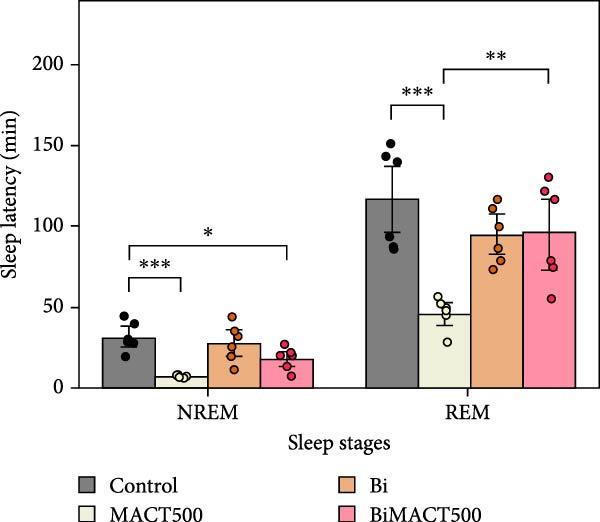
(A)

**Figure:**
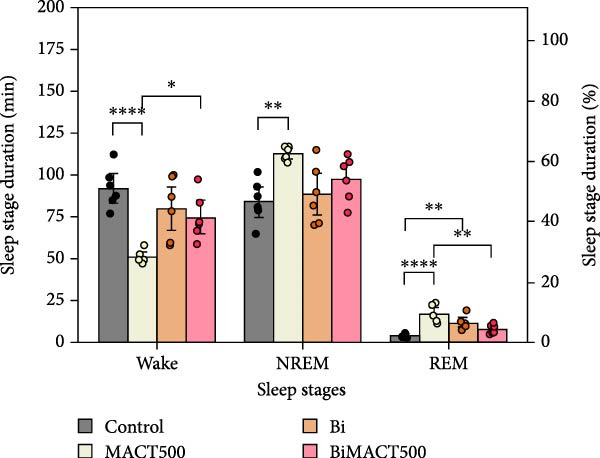
(B)

**Figure:**
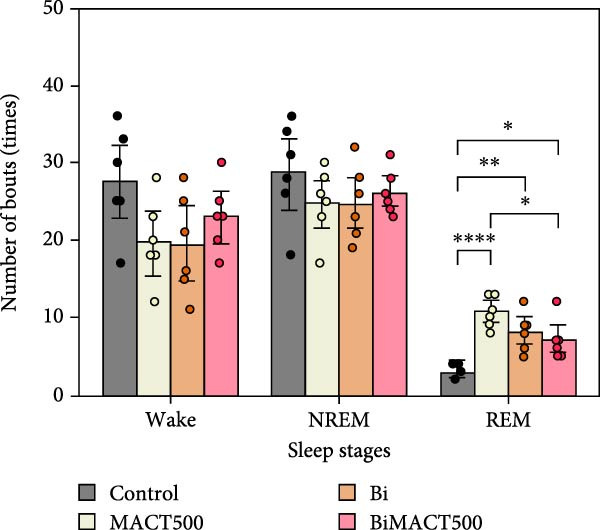
(C)

**Figure:**
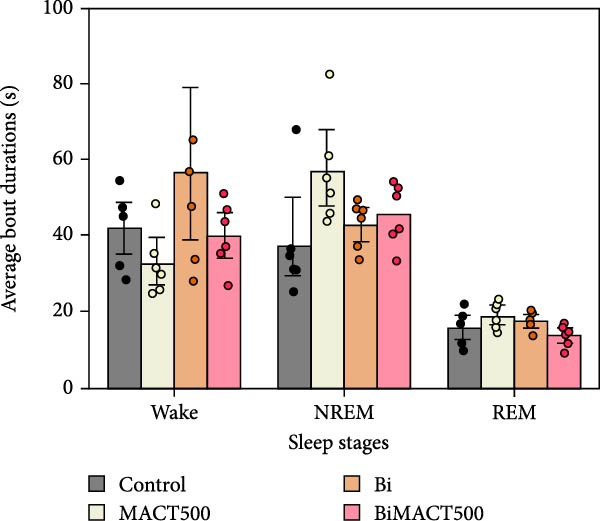
(D)

#### 3.4.2. NOR Test

Following the one‐way ANOVA analysis, we observed significant increases in the NOPI value of MACT500 rats (Table S5). However, this parameter was reversed by pretreatment with Bi (BiMACT500) (*p* = 0.0025). Administration of Bi alone exhibited no significant changes in NOPI value (Figure 7A). Additionally, considering the locomotor parameters, as seen in the total distance moved (Figure 7B) and average speed of animal movement (Figure 7C), it suggests that the NORI modulation from this experiment may not be affected by abnormalities in animal movements.

Figure 7Parameters related to neurocognition were obtained from novel object recognition tests, including the novel object preference index (NOPI) (A), total distance moved (B), and speed of movement (C), following administration of MACT under GABA_A_ receptor‐dependent conditions. *p*‐Values of  ^∗^,  ^∗∗∗^, and  ^∗∗∗∗^ are <0.05, <0.001, and <0.0001, respectively, based on one‐way ANOVA analysis followed by Tukey’s post hoc test.

**Figure:**
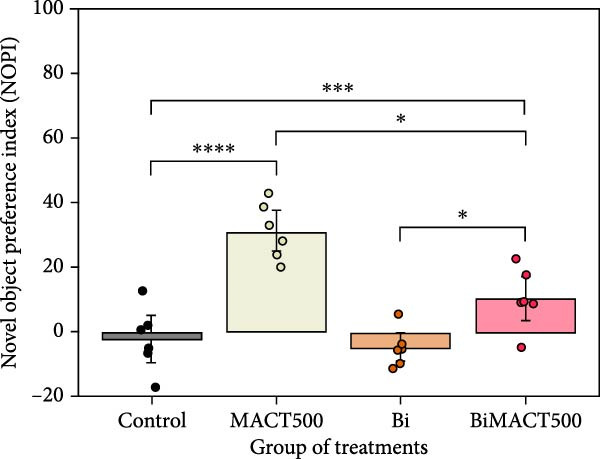
(A)

**Figure:**
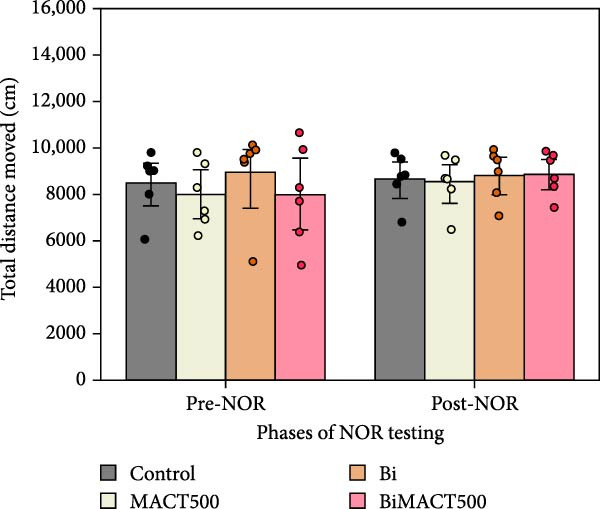
(B)

**Figure:**
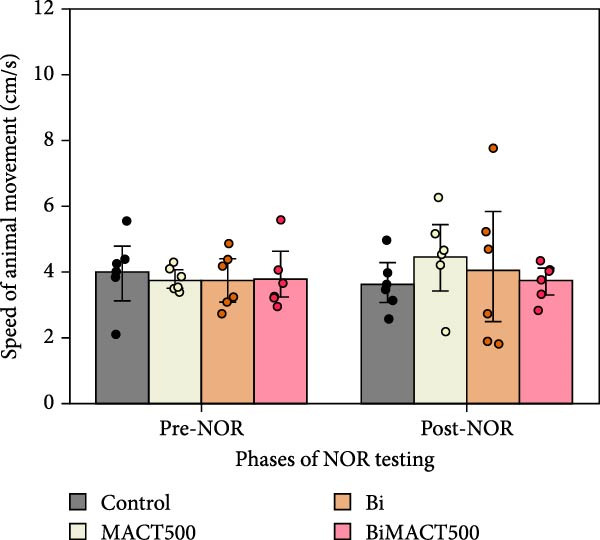
(C)

#### 3.4.3. EPM Test

According to the significant differences in anxiety status expressed as a percentage of time spent and time entries in the open arms (Table S5), it was shown that while all parameters were elevated by the MACT500 application (*p* = 0.0014 and *p* < 0.0001 for the percentage of time spent and time entries in the open arms, respectively), significant reversion was detected in MACT500 rats with Bi pretreatment (*p* = 0.0399 and *p* = 0.0357 for the percentage of time spent and time entries in the open arms, respectively; Figure 8A,B).

Figure 8Anxiolytic parameters obtained from elevated plus maze tests, including time spent in open arms (A) and the number of entries to open arms (B), were assessed following administration of MACT in GABA_A_ receptor‐dependent conditions.  ^∗^ *p* < 0.05,  ^∗∗^ *p* < 0.01,  ^∗∗∗^ *p* < 0.001,  ^∗∗∗∗^ *p* < 0.0001 indicate significance levels under one‐way ANOVA analysis followed by Tukey’s post hoc test.

**Figure:**
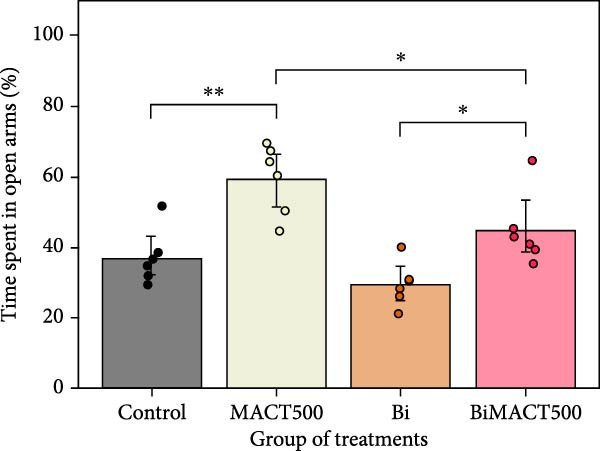
(A)

**Figure:**
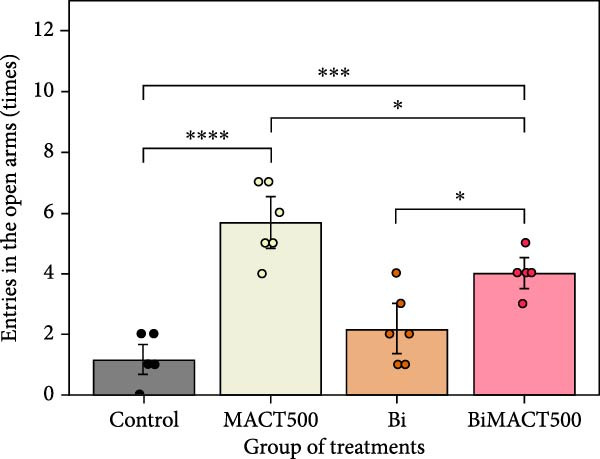
(B)

### 3.5. The Similar/Different Degrees of Brain Modulatory Effects of Plant Extracts

High‐dimensional parameters of sleep microarchitectures, NOR, and EPM tests were analyzed simultaneously using the *t*‐SNE algorithm to visualize them in a two‐dimensional projection for more straightforward interpretation. This algorithm is based on the concept of nonlinear dimensionality reduction, which separates scatter points by their similarity rather than by linear phenomena [45], as is typically observed following dose‐dependent effects. Hence, applying the *t*‐SNE algorithm for data visualization would decrease the bias affected by the drug in a dose‐dependent manner. Each point in the *t*‐SNE projection represents the probabilistic features of each rat, with those located closer to each other being more similar [38]. The qualitative results displayed a clear separation of drug treatment groups from the control group at varying degrees (Figure 9A). Quantitative analyses were applied to confirm this observation, expressed as MMD distance, to demonstrate a significantly different pattern of data distribution (F[7, 40] = 18.9907, *p*  < 0.0001). It was found that MACT500 produced a unique feature located at the farthest distance from the control group (*p* < 0.0001). However, this effect was diminished with Bi pretreatment, as observed in BiMACT500 rats (*p* < 0.0001), indicating that the beneficial impact of MACT500 preferentially mediates through a GABA_A_ receptor‐dependent mechanism. Moreover, among MACT, MA, and CT at the same concentration, the last two forms of plant extracts clearly showed their characteristics closer to control than that of MACT500 (*p* = 0.0090, and *p* = 0.0150 for MA500 and CT500 groups, respectively) (Figure 9B), suggesting a higher therapeutic efficacy of MACT500 compared to those of the individual plant materials.

Figure 9The *t*‐SNE projection mapped the significant features of monitored sedative‐hypnotic, cognitive, and anxiolytic effects of the tested compounds onto two meaningful axes (A). The centroid of the reduced data from each group was calculated and used as a reference landmark for determining maximum mean discrepancy (MMD) distance to quantitatively assess the degree of similarity or difference among the tested substances (B).  ^∗^ *p* < 0.05,  ^∗∗^ *p* < 0.01,  ^∗∗∗^ *p* < 0.001,  ^∗∗∗∗^ *p* < 0.0001 under one‐way ANOVA analysis followed by Tukey’s post hoc test.

**Figure:**
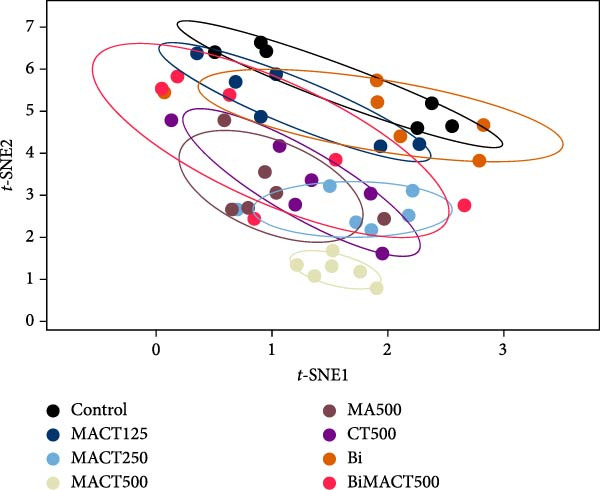
(A)

**Figure:**
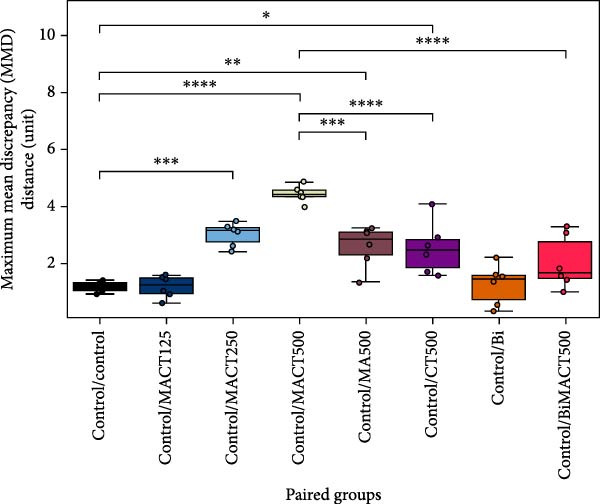
(B)

## 4. Discussion

This study highlighted the positive neuromodulation of the GABA_A_ receptor by a novel functional ingredient derived from MA combined with CT. Three core neurophysiological improvements, including sleep quality, neurocognition, and anxiolytic function, were clearly identified as key findings, which were postulated to be mediated through GABA_A_ receptor‐dependent mechanisms.

It has been consistently documented and supported by recent publications that various compounds have been chemically characterized from MA and CT. Moreover, in addition to ATC derivatives, for example, C3G, C3S, and M3G, which are commonly found in CT, these flowers also accumulated many other phenolics and flavonoids, such as GAL, QUE, KAF, rutin, chlorogenic acid, and ellagic acid [21, 34]. Some of these compounds, such as chlorogenic acid, were either additionally undetected or only exclusively observed in high amounts in MA [19]. Therefore, due to the diverse chemical compositions seen in these plants, these would contribute to having a specific interactive pattern within sources of extracts, leading to demonstrating their highly desirable outcomes and unique biological functions compared to those of individual pure compounds [47]. Hence, crude extracts were preferable in this experiment. In line with previous evidence, cognitive, anxiolytic, and sleep quality‐improving properties were seen following applications with either MA [16, 17, 48] or CT [25, 26, 49].

Accumulated data have shown that MA and its main variety of chemical compound derivatives are associated with health‐promoting functions and may have therapeutic applications mediated through various underlying mechanisms, such as controlling 5‐HT receptors and stress hormone production via manipulating the hypothalamic–pituitary–adrenal (HPA) axis [18, 19, 50]. In addition, researchers provided evidence that QUE, rutin, and MA are recognized as potent free radical scavengers and GABAergic enhancers by increasing the metabolic rates of antioxidant enzymes, for example, superoxide dismutase (SOD), catalase, glutathione peroxidase [51–53], and suppressing the activity of GABA‐metabolizing enzymes, for example, GABA‐T and succinic semialdehyde dehydrogenase (SSADH) [14, 15], respectively. These actions would enable MA to collectively exhibit elevated cognitive functions, improved anxiolytic status, and enhanced sleep quality, as seen in previous findings [16–18] and in this experiment.

Various health benefits of CT were seen due to the abundant accumulation of various ATC derivatives. It is in agreement with previous data that the key compounds mainly seen in CT belong to C3G, C3S, and M3G [20, 21]. The specific targets in the brain that responded to these molecules are still debated. Cyanidin and malvidin are proposed to enhance brain activity through several mechanisms, including reducing oxidative stress and neuroinflammation, as well as promoting biomolecules related to GABA‐dependent functions [22, 23].

According to the discussion mentioned above, deploying MA and CT as an alternative source for neuromodulatory benefits would be strong evidence. Nevertheless, based on pathophysiology backgrounds, both visible and invisible symptoms of many diseases are thought to be linked to diverse factors and complications [24, 54]. To achieve highly effective treatment dealing with these problems, drugs in polyherbal formulation forms, which may interact on multiple targets simultaneously, would provide a thorough relief [10]. This supportive data clearly explain why combinatorial ingredients at an appropriate ratio displayed more positive superior outcomes than those of any plant extract per se, as documented by consistent findings [11, 12], and this experiment of MACT‐treated conditions for both in vitro and in vivo experiments. We believed that the beneficial effect of MACT might be related to its phytochemical substances [55–57]. Even though the specific reagent found in MACT that may play a crucial role in producing higher therapeutic results is still controversial, and the identification of the exact active ingredient was outside the scope of this study. After performing the HPLC analysis, the results may offer more insights into this perspective, likely exposing rutin, C3S, and KAF activities as key contributors due to their high accumulation at concentrations of 0.266, 0.332, and 0.037 mg/g extract, respectively. By integrating these pharmacological insights with our experimental findings, this would provide a clearer understanding of how the active constituents in the extract may underlie the observed behavioral and neurophysiological outcomes as seen in the recent publications [12, 29, 35].

Among many other bioactive chemicals found in MACT, rutin and C3S are the first two most abundantly detected. Rutin, a natural bioflavonoid compound, can optimize neuronal activity in the CNS by normalizing HPA axis function and oxidative stress‐induced neuroinflammatory status [58] as well as suppressing acetylcholinesterase and GABAergic shunt enzymes, that is, GABA‐T and SSADH [14, 55]. Thus, researchers have recognized rutin as a potential neuromodulator and neuroprotector with therapeutic applications in various neurological disorders, such as improving sleep‐related parameters, depression, anxiety, learning, and memory activity [55, 58, 59].

For C3S, its effect on CNS functions remained elusive. Up‐to‐date, C3S was documented to be a neuroprotector through an in vitro study of paraquat‐induced Parkinson’s disease [60]. Previously, QUE interacting with C3S produced a synergistic inhibitory effect on the functions of the blood–brain barrier (BBB) expressing ABCB1 protein [57]. However, QUE was proven to have inferior effects to KAF on many organs’ functioning [56, 61, 62]. Therefore, the MACT extract, which exhibits an exclusive high accumulation of KAF along with C3S, may probably synergize to sensitize brain functioning responses through ABCB1 protein‐dependent activities more rapidly. Additionally, in the form of crude extract, C3S‐rich extract from CT combined with *Garcinia mangostana* was also found to produce synergistic effects in vitro biological activities [63].

Even if KAF was observed in MACT in a relatively low amount compared to those of the first two compounds, it was postulated to partly facilitate MACT in demonstrating high beneficial results, as KAF was exceptionally highest among the three forms of plant extracts. KAF is a type of natural flavonoid that has a positive influence on various brain functions [64]. Interestingly, it was distinctively documented that KAF appeared to exert the most superior modulatory activities than those of many other flavonoids in both peripheral [54, 61, 65, 66] and central organs [56, 62]. This is likely due to the presence of one –OH moiety on the benzene ring of the flavone core structure, which would make KAF more lipophilic and less likely to form hydrogen bonds with water molecules [67]. In addition, KAF is a nonplanar structure, which allows for more conformational flexibility and increased penetration through the BBB and plasma membrane [68]. These could eventually enable KAF to be a more rapid manifestation of both central and peripheral effects. However, KAF in free form could have potential limitations in bioavailability due to its low water solubility and undergoes extensive first‐pass metabolism in the gut and liver [69]. On the other hand, KAF in conjugated form minimizes undesirable effects on normal cells [70]. These forms are likely to protect the active form of KAF from early degradation, thereby extending its circulation time. In addition, conjugating KAF with hydrophilic carriers, such as various ATC derivatives discussed in MACT500, could improve solubility and absorption. This approach may also utilize carrier‐mediated transport systems to cross membranes more effectively [71]. Altogether, this suggests that the MACT, which contains the three main candidate compounds—rutin, C3S, and KAF—along with numerous unidentified phytochemicals, might offer greater neuromodulatory benefits in MACT500‐treated rats compared to other formulations.

Aside from those three main compounds that were specifically focused on, it is undeniable that other compounds seen in the HPLC unidentified peaks would be recognized as potential contributors. It is likely to be due to many other substances presented in CT, for example, delphinidin and petunidin [24, 34], and MA, for example, GABA and morin [19, 72] could also exhibit a positive result of MACT500 through complex interactions, as they were found to play a significant role in CNS improvements in previous findings [19, 73, 74]. In addition, the prevention of anxiogenic behavior and memory deficit in animals, as well as the promotion of sleep quality in humans, was accompanied by the application of these chemical constituents, as they were evidenced to enhance GABAergic transmission via allosteric or direct regulation of the GABA_A_ receptor [19, 24, 26, 75].

The following experiment revealed the novel finding of a potent MACT500 on meditating neurophysiological and mental improvements through a GABA_A_ receptor‐dependent mechanism. GABAergic transmission is well‐known as a central driver of neuronal inhibition through the activation of GABA_A_ and GABA_B_ receptors, resulting in hyperpolarization. However, due to the rapid synaptic responses of the ionotropic GABA_A_ receptor, this target is preferable for sedative‐hypnotic drug development [76]. Hence, the reduction of wakefulness and latency to fall asleep, along with the increase in sleep time and total sleep episodes, was seen as a general physiological response following GABA_A_ receptor upregulation [16, 19]. Dose‐dependent effects of NREM and REM sleep are clearly evident in experiment 1 following administration of MACT. In sleep medicine, NREM and REM sleep are mainly directly manifested by modulating acetylcholine and monoamine, working together with GABAergic neurotransmitters in a different functioning ratio [77], and REM sleep seems to be preferably controlled by GABAergic functions [35]. Therefore, we speculated that MACT may have a higher tendency to interact with GABAergic systems, as a majority of parameters related to MACT500‐induced REM sleep generation were significantly antagonized by Bi pretreatment. Interestingly, while both allosteric interaction at the BZs recognition site on GABA_A_ receptors and direct activation of the active site of this receptor appeared to play a crucial role in the initiation and maintenance of NREM sleep, the manifestation of REM sleep seems to be controlled exclusively by the significant function of the latter mechanism [78]. Overall, these results suggest that MACT500 is more effective as a GABAergic enhancer, specifically acting as a GABA_A_ receptor agonist to promote increased REM sleep. In addition, in rats treated with BiMACT500, an increase in wakefulness was observed, accompanied by a decrease in REM sleep duration and the number of REM sleep bouts, without affecting NREM sleep parameters. This pattern may indicate that MACT500 facilitates a unique sleep/wake cycle transition, where the wake phase passes through a brief NREM sleep stage before entering a prolonged REM sleep period.

Although REM sleep mainly involves GABAergic mechanisms, as mentioned earlier, and this was the specific focus in this experiment, the influence of MACT containing diverse ATCs and flavonoids on REM sleep, through modulation of serotonergic, dopaminergic, or cholinergic systems, cannot be overlooked, as shown in earlier research [29]. Alterations in sleep patterns, as well as cognitive function and anxiolytic status, have been widely recognized to result from the functioning of these neurotransmitter systems in a particular manner [12, 19, 23]. However, the exact mechanism of this perspective remains to be elucidated in future experiments.

Various neurotransmitter systems, that is, GABA, monoamine, and acetylcholine, have been well documented to play a significant role in nonspatial memory and anxiolytic effects based on NOR and EPM test assessment, respectively [11, 12, 79]. In addition to REM sleep, the GABA_A_ receptor is also of pharmacologically considerable importance in the expression of cognitive and anxiolytic behaviors following the administration of either synthetic or natural compounds [79, 80] as these behaviors are reversed depending on the application with GABA_A_ receptor antagonist [80, 81]. Although the exact mechanisms of the GABA_A_ receptor underlying these perspectives are still debated so far. Theoretically, it was postulated that GABA seems to act as a circuit pacemaker, driving many other brain components to function more appropriately and integratively [82]. The fast‐spiking GABAergic interneurons were found to synchronize network activity, facilitating oscillations across various frequency bands. Locally, this kind of cortical interneurons‐generated gamma rhythm is of significant interest for the shaping and understanding of various cognitive processes [83]. In humans, the processing of cortical information was detected concomitantly with distinct gamma and alpha activity [84]. On the other hand, working memory deficits appeared to be linked to dysfunctional gamma oscillations in the prefrontal cortex (PFC) of schizophrenia patients [85], as well as following the blockade of PFC GABA_A_ receptors in rats [86]. These critical functions would also contribute to anxiolytic status, as the common mechanisms of memory‐anxiety interrelationship were discussed previously [3, 4, 87]. In addition to controlling neuronal synchrony within the microcircuitry, GABAergic activity would also affect synchrony across targets for long‐distance connections. It has been suggested that long‐range GABAergic neurons are essential for the temporal coordination of neuronal activity across numerous remote brain regions, which is necessary to improve thinking and cognitive functions [88]. For instance, GABAergic neurons, which reciprocally link the hippocampus and PFC, have been depicted to have top‐down regulation via GABA‐mediated recurrent inhibition, allowing for the demonstration of a potential function in cognitive activity [89].

Although the association between GABAergic transmission and/or GABA_A_ receptors, which mediate various neurophysiological functions, was discussed extensively in the previous section, the deployment of GABA_A_ receptors as a crucial target for drug development remains to be fully elucidated. It is due to the different stoichiometry of the GABA_A_ receptor subunits, and the activation of this receptor can be achieved through various endogenous and exogenous substances in response to a unique binding site, such as neurosteroids, muscimol, ethanol, barbiturates, BZs, Bi, and GABA [76]. Consequently, different ligand reactivities enabling various diverse behavioral responses are also observed accordingly. Previously, the mediation through *α*_2_‐containing GABA_A_ receptors successfully exerted keen anxiolytic properties while avoiding undesired side effects [90]. On the other hand, the BZs, for instance, are known to produce detrimental effects on motivation, motor coordination, and cognition by mediating dependently on other *α* GABA_A_ receptor subunits [91]. Thus, we speculated that MACT500 would primarily selectively bind to *α*_2_‐containing GABA_A_ receptors, as no defective locomotor function, memory dysfunction, or anxiogenic behavior was observed in our findings. A multiline of evidence also consistently supported our results that using natural compounds seems to be more satisfying and preferable in mediating cognitive and anxiolytic activities through pharmacological manipulation of the GABA_A_ receptor‐dependent mechanism without displaying the BZs‐like side effects [79, 80].

Although the pharmacological properties of MACT500 have been documented and discussed, it is undeniable that MACT500 indirectly enhances neurocognition and anxiolytic behavior by promoting higher sleep quality following drug administration. Previous research proposed that REM sleep has been thought to regulate dreaming, enhance emotions, and promote cognitive and memory function, as many genes, proteins, and neurotransmitters required for memory consolidation, emotional regulation, and neuronal plasticity appear to be upregulated, specifically in brain tissue during this sleep state [92–94]. Additionally, a positive interrelationship between REM sleep quality and emotional memory processing was also seen in this experiment, aligned with a previous publication [95]. The top view of these discussions eventually indicated that MACT500 may serve as a memory enhancer and anxiolytic agent indirectly, through prolonged REM sleep‐induced improvement effects, and/or directly, through its pharmacological interaction. However, pharmacokinetic and pharmacodynamic studies of MACT500 are required for additional confirmation from this perspective.

Although in vivo oxidative stress markers such as malondialdehyde, SOD, and catalase levels in the brain were not measured to validate many positive results, previous studies have generally reported antioxidant activity associated with improvements in sleep and mental health [12, 96]. Therefore, the positive outcomes observed here are likely partly due to the high oxidant scavenging capabilities of the MACT formula, as shown in the in vitro tests, and align with recent research finding [29].

Numerous studies have shown that the neuropharmacological benefits of herbal mixtures arise from modulating neurotransmitter functions and reducing oxidative stress [11, 12, 29]. These effects result from the complex interactions among various bioactive compounds in these herbs, producing a synergistic effect. While the exact mechanisms—especially regarding polyphenols—are still unclear, this synergy may involve altering the pharmacokinetics and pharmacodynamics of active substances, leading to beneficial metabolites that help restore neurotransmitter balance, such as GABA, and decrease oxidative stress. Ultimately, this enhances sleep quality and mental health in both healthy and pathological conditions, as demonstrated in this study and supported by prior researches [12, 29].

The Bi agent was used in this experiment to antagonize the possible effects of MACT500‐induced neuronal hyperpolarization mediated at the active site of the GABA_A_ receptor, thereby promoting Cl^−^ influx [8]. Hence, neuronal depolarization and excitation were seen following Bi application. Additionally, Bi was also found to inhibit the activity of small‐conductance Ca^2+^‐activated K^+^ (SK) channels, the primary mediator of the medium after hyperpolarization, which is typically expressed in the interneurons [97]. Blocking this channel type potentially enhances neural firing rate, sympathetic outflow, and alertness, respectively [98]. Surprisingly, it significantly elevated specific parameters of REM sleep, including REM sleep duration and the number of REM sleep bouts. Previous evidence has demonstrated that, aside from the GABA neurotransmitter system, REM sleep is also regulated by cholinergic function, primarily originating from cholinergic REM‐on neurons in the brainstem. These could be supported by evidence that GABA_A_ receptors located on cholinergic boutons in the pontine reticular formation are responsible for the REM sleep induction by GABA_A_ receptor antagonists, Bi, through blocking GABA inhibition of acetylcholine release [99]. Owing to this evidence, therefore, Bi’s role in combination therapy may not depend solely on GABA_A_ antagonism, as multiple neurotransmitter systems are involved, as indicated by previous research. It was proposed that MACT could alleviate insomnia by inhibiting acetylcholinesterase and GABA‐T activities [29]. Our findings indicated that all REM sleep parameters affected by MACT were antagonized following Bi pretreatment, suggesting that MACT’s beneficial effects are mainly mediated via a GABA_A_ receptor‐dependent mechanism and, to some extent, through cholinergic transmission. Based on this evidence, the dual regulation of REM sleep by the GABAergic and cholinergic systems following MACT500 treatment is plausible and remains to be confirmed with further investigation.

After completing 3 h of sleep profile monitoring in this experiment, the animals underwent behavioral testing, including the NOR and EPM tests. The half‐life of the Bi after oral administration is approximately 1.6 h [100]. Hence, a potent Bi may be dramatically converted into its metabolized form, bicucine, which is a very weak antagonist of the GABA_A_ receptor [101]. Interestingly, Bi, in combination with MACT500, continues to effectively antagonize the properties of MACT500. Many lines of evidence also support these findings that using Bi in low concentration should be mixed or co‐administered with other molecules to reduce the production rate of its metabolized compounds for prolonging its pharmacological effects centrally [3, 4]. Hence, the deployment of Bi *per se* for intraperitoneal injection was recommended at a high dose treatment (6.0–8.0 mg/kg) [102]. These collectively explain why animals in the Bi administration in the Bi and BiMACT500 groups exhibited a majority of sleep profile and animal behavior parameters in a comparable manner and in a remaining‐GABA_A_‐receptor‐antagonized function, respectively.

Although the current study presents novel findings, several limitations should be considered in future research to understand better how the GABA_A_ receptor influences sleep microarchitecture, cognitive function, and anxiolytic behavior. First, conducting a radioligand binding assay would provide a precise measurement of the ligand–receptor interaction affinity. Second, without a standard reference drug, it becomes harder to assess whether the effects of MACT are better, comparable, or worse than existing results. This also complicates the ability to apply the findings clinically or to compare them with other studies that employ standard drugs.

Finally, all pieces of evidence clearly indicate that MACT500 exhibits positive, unique patterns, leading to improvements in many aspects of CNS function. However, the higher‐dimensional effects following MACT500 use remain largely unexplored. To clarify the pathological features after chronic treatment and employ molecular techniques to investigate the modulation of related molecules in the brain is also urgent. Furthermore, based on the abnormalities in GABAergic transmission in animals with insomnia and autism [12, 103], a thorough investigation into the ameliorative effects of MACT500 in animal models with insomnia and autism is also essential for validation.

## 5. Conclusion

**Figure 10:**
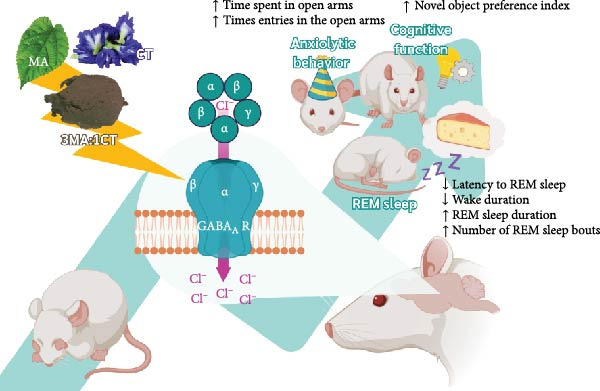
The mechanisms through which MACT500 enhances neurological responses, such as sleep, cognitive function, and anxiolytic effects, are primarily mediated by GABA_A_ receptor‐dependent pathways in rats (illustration created with BioRender.com).

In summary, the functional ingredients from MACT have been well‐documented through in vitro and in vivo studies to deliver better outcomes than MA or CT alone. These include improved REM sleep, enhanced cognitive function, and reduced anxiety, all of which are achieved through GABA_A_ receptor modulation, as shown in the proposed mechanisms in Figure 10. Such results could lead to the development of MACT as a new drug or food supplement, in the form of a polyherbal formulation designed to enhance brain function and improve mood. Given the positive effects involving the GABA_A_ receptor pathway, further research—such as animal studies on GABA_A_ receptor‐related conditions or human trials for issues, such as insomnia or autism—may be required to confirm MACT’s efficacy.

**Nomenclature**
BBB: Blood–brain barrier
Bi: Bicuculline methiodide
BZs: Benzodiazepines
C3G: Cyanidin‐3‐glucoside
C3S: Cyanidin‐3‐sophoroside
CT: Butterfly pea flowers
EEG: Electroencephalogram
EMG: Electromyogram
EPM: Elevated plus‐maze
GABA‐T: GABA transaminase
GAL: Gallic acid
HPA: Hypothalamic‐pituitary‐adrenal axis
KAF: Kaempferol
M3G: Malvidin‐3‐glucoside
MA: Mulberry leaves
MACT: Combination of 3MA1CT ratio
MMD: Maximum mean discrepancy
NOPI: Novel object preference index
NOR: Novel object recognition
NREM: Non‐rapid eye movement sleep
PC: Parietal cortex
PFC: Prefrontal cortex
PSD: Power spectral density
QUE: Quercetin
REM: Rapid eye movement sleep
SSADH: Succinic semialdehyde dehydrogenase
TACs: Total anthocyanin contents
TFCs: Total flavonoid contents
TPCs: Total phenolic contents.

## Disclosure

The final version of the manuscript was thoroughly reviewed and approved by all the authors.

## Conflicts of Interest

The authors declare no conflicts of interest.

## Author Contributions

**Jakkrit Nukitram:** project administration, methodology, validation, formal analysis, investigation, resources, data curation, writing – original draft, visualization, funding acquisition, conceptualization. **Aonvara Kanjanavattana**: formal analysis. **Panlekha Rungruang and Nobuhiro Zaima**: software. **Dania Cheaha**: methodology. **Nattaporn Yotyatthai, Pannita Kaewudom, Pichayapa Promkasikorn, Patharakan Kaowsuwan, and Wipawee Thukhammee**: data curation. **Jintanaporn Wattanathorn**: writing – review and editing, supervision.

## Funding

This research is financially supported by the Young Researcher Development Project of Khon Kaen University Year 2024 (Grant AW 660201.1.10.1/W2274) and the Research, Innovation, and Academic Services Fund, Faculty of Science, Khon Kaen University Fiscal Year 2024 (Grant SC102/2023).

## Supporting Information

Additional supporting information can be found online in the Supporting Information section.

## Supporting information


**Supporting Information** Figure S1. The workflow diagram for analyzing sleep microarchitecture is depicted above. Briefly, raw EEG and EMG data were first down‐sampled from 2 to 200 Hz. It was then reorganized, with the first and second channels assigned to the EEG and EMG, respectively. The preprocessed data were passed through the sliding_window function (YASA package, version 0.6.3) [41] with a 5‐s window to segment the signals for rapid analysis. These smaller segments were continuously input into the fast Fourier transform algorithm using Welch’s method (welch command of SciPy package, version 1.11.4) to compute the PSD across the delta (0.5–4.0 Hz), theta (4.0–8.0 Hz), alpha (8.0–12.0 Hz), beta (12.0–30.0 Hz), gamma I (30.0–45.0 Hz), and gamma II (60.0–95.0 Hz) frequency ranges (step 1 by using the attached file 0001) [39]. A blank file was generated, containing dummy segments in equal amounts, with the length of the data recording, to serve as a saving file for the results from the AccuSleep toolbox (step 2 by using the attached file 0002). The conversion file type was then changed from .edf (from the recording machine) to .mat (readable by the AccuSleep toolbox) (step 3 by using the attached file 0003). After finishing the data preprocessing process, the PSD in two data channels was automatically scored for sleep/wake stages using the AccuSleep toolbox (MATLAB version R2023a) with a setting of 5‐s epoch length and 200 Hz sampling rate (step 4). The protocol for processing this step had existed in the original publication of the toolbox [42]. Subsequently, Python code for multitaper spectrogram analysis [43] and hypnogram visualization (YASA package, version 0.6.3) [41] was applied to display changes in the spectrogram from the EEG and EMG alongside extracting the important parameters for the sleep profile for statistical analysis (step 5 by using the attached file 0004). Table S1. Phytochemical contents (TPCs, TFCs, and TACs) and biological activities (FRAP, DPPH, and GABA‐T suppression) of various combinations between MA and CT. Data are presented as mean ± S.E.M (carried out in triplicate). Figure S2. The HPLC fingerprint of plant extracts was analyzed as follows: MACT (at a concentration of 40 mg/mL) and CT (at a concentration of 50 mg/mL) are represented by the gray (A2) and blue (A3) lines, respectively, compared to the red line of standard GAL (at a concentration of 80 μg/mL) detected at 275 nm (A1). The red (B1), gray (B2), and blue (B3) lines represent the standard chlorogenic acid (at 100 μg/mL), MA (at 50 mg/mL), and MACT (at 40 mg/mL), respectively, detected at 320 nm. The red (C1), gray (C2), blue (C3), and dark red (C4) lines represent the detection at 254 nm for standard ellagic acid (at 40 μg/mL), MA (at 50 mg/mL), MACT (at 40 mg/mL), and CT (at 50 mg/mL), respectively. Finally, the red line (D1) for standard rutin, QUE, and KAF (at 40 μg/mL) detected at 370 nm (D1) aligns with the gray (D2), blue (D3), and dark red (D4) lines of MA (at 50 mg/mL), MACT (at 40 mg/mL), and CT (at 50 mg/mL) for quantification. Table S2. HPLC analysis of the phenolic/flavonoid contents in the MACT, MA, and CT. Data are presented as mean ± SD (carried out in triplicate). Figure S3. Chromatogram of plant extracts. Red line (1) was the standards of the anthocyanin, including delphinidin‐3‐glucoside (Del3G), cyanidin‐3‐sophoroside (C3S), cyanidin‐3‐glucoside (C3G), cyanidin‐3‐rutinoside (C3R), petunidin‐3‐O‐beta‐D‐glucoside (Pet3G), pelargonidin‐3‐glucoside (Pel3G), peonidin‐3‐O‐glucoside (Peo3G), and malvidin‐3‐glucoside (M3G), respectively (at 5 μg/mL). The gray (2) and blue (3) lines were the MACT (at 40 mg/mL), and CT (at 20 mg/mL) were detected at 530 nm. Table S3. HPLC analysis of the anthocyanin contents in the MACT, MA, and CT. Data are presented as mean ± SD (carried out in triplicate). Table S4. Significant parameters of sedative‐hypnotic, cognitive, and nootropic effects of MACT in a dose‐dependent fashion and in comparison, to either MA or CT alone. Data are presented as mean ± S.E.M. (*n* = 6/group). Table S5. Significant parameters of sedative‐hypnotic, cognitive and anxiolytic effects of MACT in GABA_A_ receptor‐dependent conditions. Data are presented as mean ± S.E.M. (*n* = 6/group).

## Data Availability

The data that supports the findings of this study are available in the supporting information section of this article.
