# Supplementary material for: A Novel Functional Ingredient Derived From a Mixture of Mulberry (Morus alba L.) Leaves and Butterfly Pea (Clitoria ternatea L.) Flowers Enhances Rapid Eye Movement Sleep, Cognitive Function, and Anxiolytic Behavior via GABAA Receptor‐Dependent Mechanism in Rats
Source: Oxid Med Cell Longev. 2026 Feb 14;2026:2305848. doi: 10.1155/omcl/2305848 (PMC12906349; doi:10.1155/omcl/2305848)
Supplement: Supplementary file 1 — Supporting Information Figure S1. The workflow diagram for analyzing sleep microarchitecture is depicted above. Briefly, raw EEG and EMG data were first down‐sampled from 2 to 200 Hz. It was then reorganized, with the first and second channels assigned to the EEG and EMG, respectively. The preprocessed data were passed through the sliding_window function (YASA package, version 0.6.3) [41] with a 5‐s window to segment the signals for rapid analysis. These smaller segments were continuously input into the fast Fourier transform algorithm using Welch’s method (welch command of SciPy package, version 1.11.4) to compute the PSD across the delta (0.5–4.0 Hz), theta (4.0–8.0 Hz), alpha (8.0–12.0 Hz), beta (12.0–30.0 Hz), gamma I (30.0–45.0 Hz), and gamma II (60.0–95.0 Hz) frequency ranges (step 1 by using the attached file 0001) [39]. A blank file was generated, containing dummy segments in equal amounts, with the length of the data recording, to serve as a saving file for the results from the AccuSleep toolbox (step 2 by using the attached file 0002). The conversion file type was then changed from .edf (from the recording machine) to .mat (readable by the AccuSleep toolbox) (step 3 by using the attached file 0003). After finishing the data preprocessing process, the PSD in two data channels was automatically scored for sleep/wake stages using the AccuSleep toolbox (MATLAB version R2023a) with a setting of 5‐s epoch length and 200 Hz sampling rate (step 4). The protocol for processing this step had existed in the original publication of the toolbox [42]. Subsequently, Python code for multitaper spectrogram analysis [43] and hypnogram visualization (YASA package, version 0.6.3) [41] was applied to display changes in the spectrogram from the EEG and EMG alongside extracting the important parameters for the sleep profile for statistical analysis (step 5 by using the attached file 0004). Table S1. Phytochemical contents (TPCs, TFCs, and TACs) and biological activities (FRAP, D [file OMCL-2026-2305848-s001.docx]

Supplement materials

**A novel functional ingredient derived from a mixture of mulberry (*Morus alba* L.) leaves and butterfly pea (*Clitoria ternatea* L.) flowers enhances rapid eye movement sleep, cognitive function, and anxiolytic behavior via GABA_A_ receptor-dependent mechanism in rats**

Jakkrit Nukitram, Aonvara Kanjanavattana, Panlekha Rungruang, Nattaporn Yotyatthai, Pannita Kaewudom, Pichayapa Promkasikorn, Patharakan Kaowsuwan, Nobuhiro Zaima, Dania Cheaha, Wipawee Thukhammee, Jintanaporn Wattanathorn


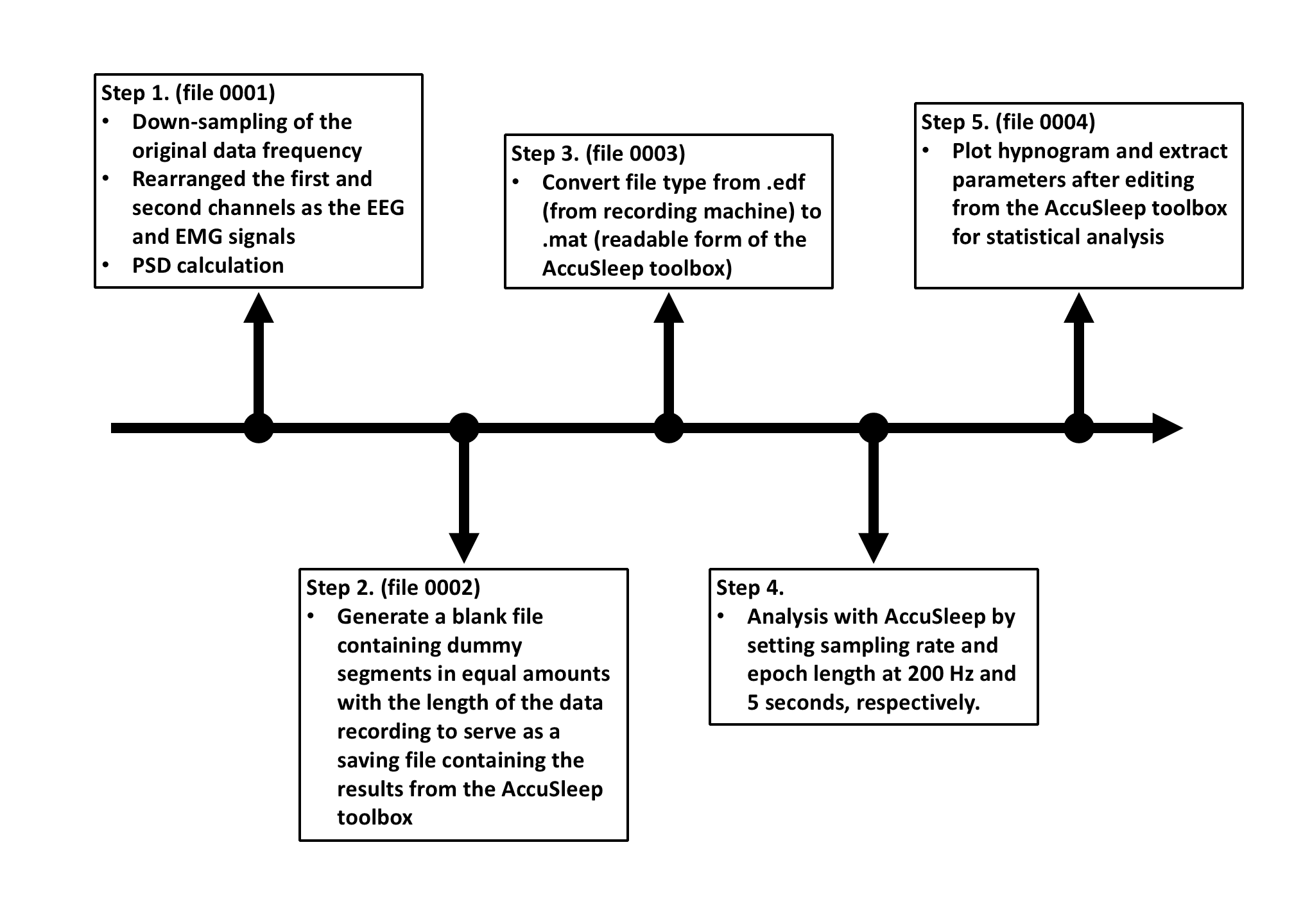


**Figure S1**

The workflow diagram for analyzing sleep microarchitecture is depicted above. Briefly, raw EEG and EMG data were first down-sampled from 2 kHz to 200 Hz. It was then reorganized, with the first and second channels assigned to the EEG and EMG, respectively. The pre-processed data was passed through the *sliding_window* function (YASA package, version 0.6.3) [1] with a 5-second window to segment the signals for rapid analysis. These smaller segments were continuously input into the fast Fourier transform algorithm using Welch’s method (welch command of SciPy package, version 1.11.4) to compute the PSD across the delta (0.5 – 4.0 Hz), theta (4.0 – 8.0 Hz), alpha (8.0 – 12.0 Hz), beta (12.0 – 30.0 Hz), gamma I (30.0 – 45.0 Hz), and gamma II (60.0 – 95.0 Hz) frequency ranges (Step 1 by using the attached file 0001) [2]. A blank file was generated, containing dummy segments in equal amounts, with the length of the data recording, to serve as a saving file for the results from the AccuSleep toolbox (Step 2 by using the attached file 0002). The conversion file type was then changed from .edf (from the recording machine) to .mat (readable by the AccuSleep toolbox) (Step 3 by using the attached file 0003). After finishing the data preprocessing process, the PSD in two data channels was automatically scored for sleep/wake stages using the AccuSleep toolbox (MATLAB version R2023a) with a setting of 5-second epoch length and 200 Hz sampling rate (Step 4). The protocol for processing this step had existed in the original publication of the toolbox [3]. Subsequently, Python code for multitaper spectrogram analysis [4] and hypnogram visualization (YASA package, version 0.6.3) [1] was applied to display changes in the spectrogram from the EEG and EMG alongside extracting the important parameters for the sleep profile for statistical analysis (Step 5 by using the attached file 0004).

**S1 Table**

Phytochemical contents (TPCs, TFCs, and TACs) and biological activities (FRAP, DPPH, and GABA-T suppression) of various combinations between MA and CT. Data are presented as mean ± S.E.M (carried out in triplicate).

| **MA: CT** | **TPCs**  **(mg GAL equivalent/g)** | **TFCs**  **(mg QUE equivalent/g)** | **TACs**  **(mg C3G equivalent/L)** | **DPPH IC_50_**  **(mg/mL)** | **FRAP IC_50_**  **(mg/mL)** | **GABA-T suppression IC_50_**  **(mg/mL)** |
| --- | --- | --- | --- | --- | --- | --- |
| 0MA: 1CT | 48.111 ± 0.424^bc^ | 11.993 ± 0.153^d^ | 5.700 ± 0.323^c^ | 5.107 ± 0.241^b^ | 2.368 ± 0.054^cd^ | 2.764 ± 0.023^b^ |
| 1MA: 0CT | 47.278 ± 0.893^bc^ | 4.752 ± 0.159^b^ | 1.380 ± 0.103^ab^ | 7.692 ± 0.083^de^ | 1.819 ± 0.028^b^ | 3.048 ± 0.014^bc^ |
| 1MA: 1CT | 47.278 ± 0.424^bc^ | 5.700 ± 0.037^c^ | 0.957 ± 0.123^ab^ | 7.106 ± 0.153^cd^ | 1.780 ± 0.034^b^ | 3.429 ± 0.018^cd^ |
| 1MA: 2CT | 43.944 ± 0.578^ab^ | 5.064 ± 0.033^b^ | 0.423 ± 0.046^a^ | 8.535 ± 0.172^e^ | 2.193 ± 0.039^c^ | 3.768 ± 0.021^d^ |
| 1MA: 3CT | 46.167 ± 0.735^bc^ | 6.050 ± 0.047^cd^ | 0.802 ± 0.111^a^ | 6.288 ± 0.087^c^ | 2.478 ± 0.031^d^ | 2.611 ± 0.041^b^ |
| 2MA: 1CT | 40.889 ± 0.893^a^ | 2.604 ± 0.019^a^ | 0.200 ± 0.022^a^ | 8.545 ± 0.033^e^ | 1.778 ± 0.024^b^ | 4.676 ± 0.219^e^ |
| 3MA: 1CT | 51.444 ± 0.578^c^ | 6.587 ± 0.040^d^ | 2.115 ± 0.207^b^ | 4.020 ± 0.048^a^ | 1.046 ± 0.007^a^ | 1.820 ± 0.013^a^ |
| F-test, *p*-value | [F(6, 14) = 8.157, *P* < 0.0001] | [F(6, 14) = 351.742, *P* < 0.0001] | [F(6, 14) = 44.534, *P* < 0.0001] | [F(6, 14) = 53.754, *P* < 0.0001] | [F(6, 14) = 67.680, *P* < 0.0001] | [F(6, 14) = 38.050, *P* < 0.0001] |

MA; mulberry leaves, CT; butterfly pea flowers, TPCs; Total phenolic contents, TFCs; Total flavonoid contents, TACs; Total anthocyanin contents, GAL; gallic acid, QUE; quercetin, C3G; cyanidin-3-glucoside, DPPH; 1,1-diphenyl-2-picryl hydrazyl radicals, FRAP; ferric-reducing antioxidant power, GABA-T; gamma-aminobutyric acid transaminase.

The statistical differences were adjusted with a one-way ANOVA followed by Tukey's post hoc correction when the *p*-value was less than 0.05. A difference in symbolic letters means statistical differences within parameters.

**(A)**


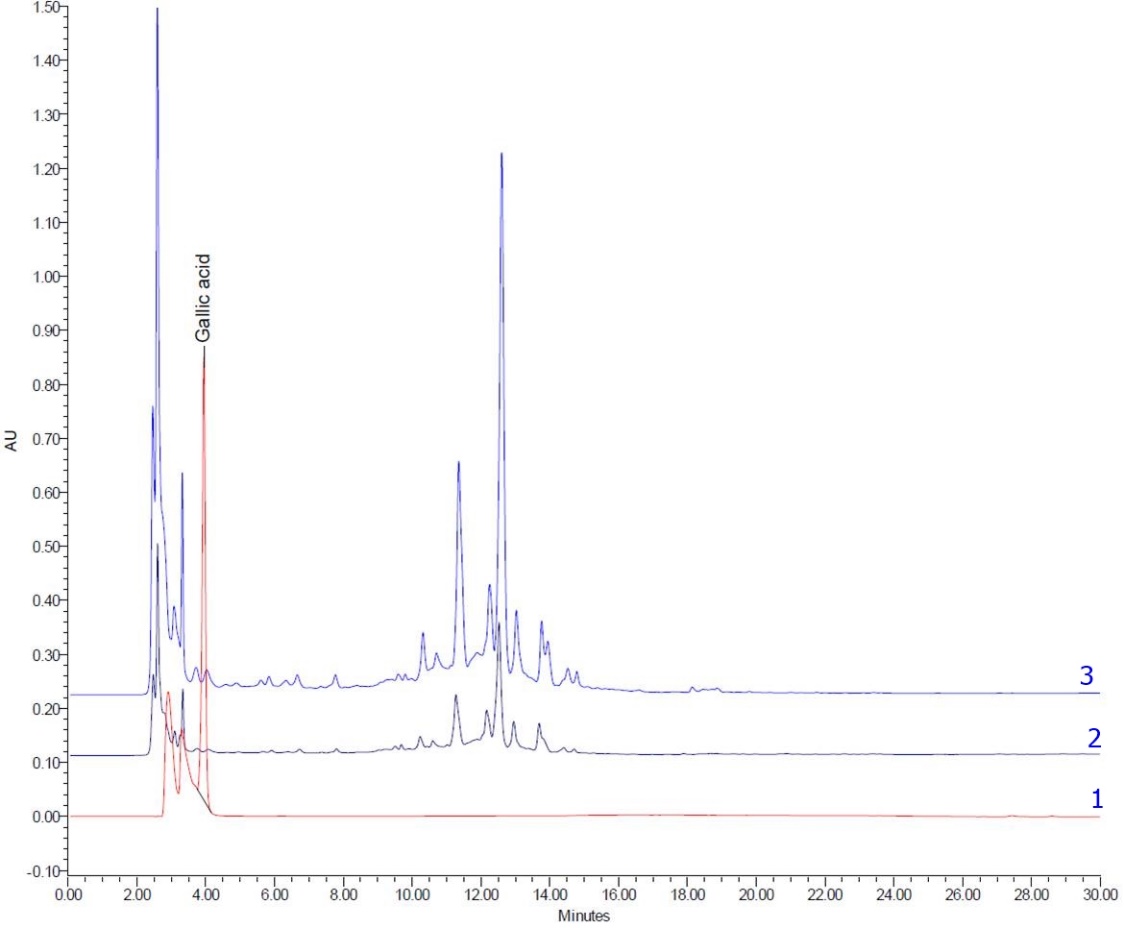

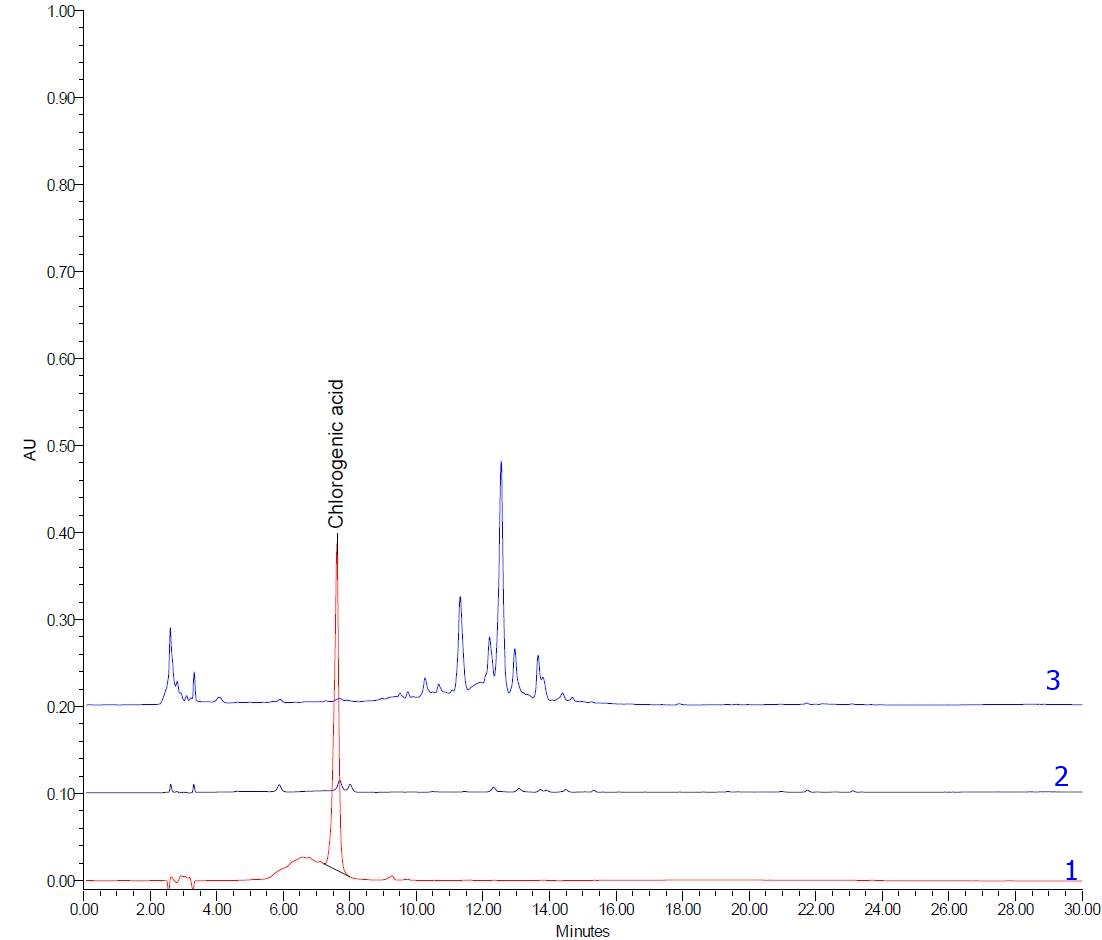


**(B)**


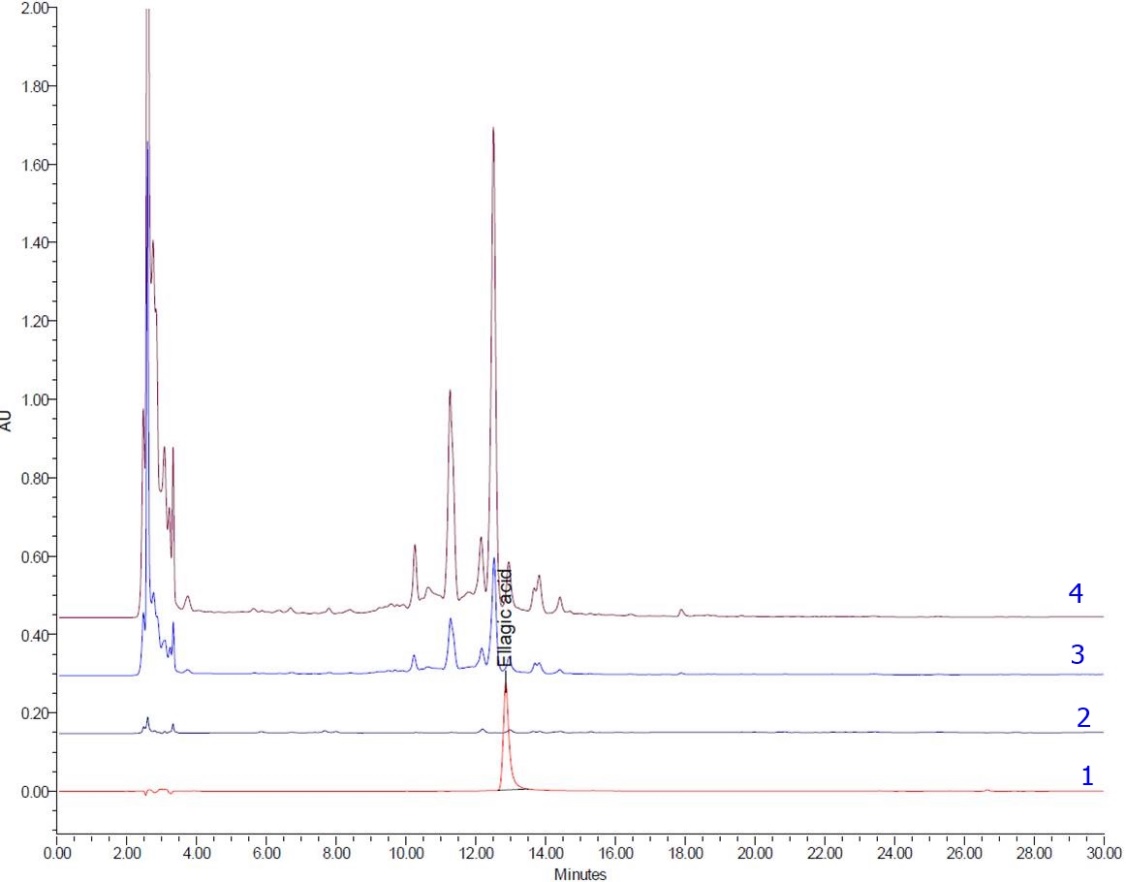


**(D)**

**(C)**


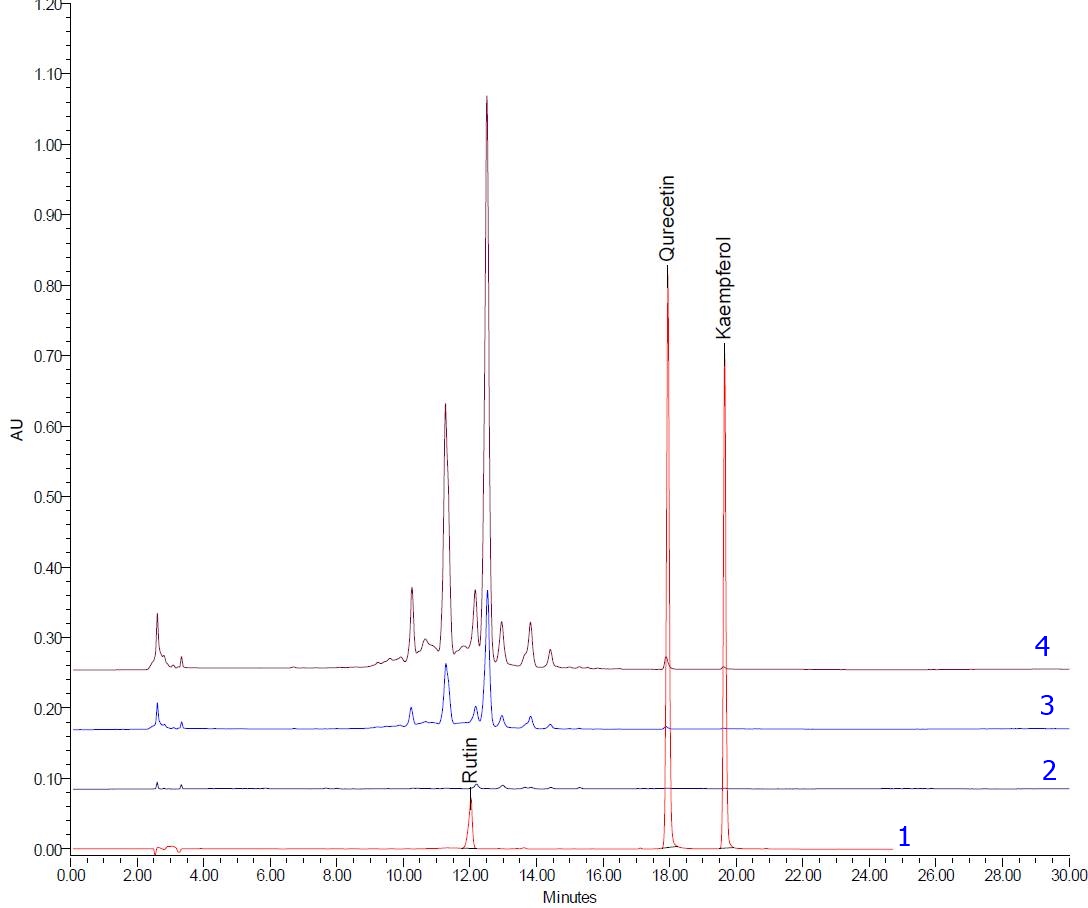


**Figure S2**

The HPLC fingerprint of plant extracts was analyzed as follows: MACT (at a concentration of 40 mg/mL) and CT (at a concentration of 50 mg/mL) are represented by the grey (A2) and blue (A3) lines, respectively, compared to the red line of standard GAL (at a concentration of 80 μg/mL) detected at 275 nm (A1). The red (B1), grey (B2), and blue (B3) lines represent the standard chlorogenic acid (at 100 μg/mL), MA (at 50 mg/mL), and MACT (at 40 mg/mL), respectively, detected at 320 nm. The red (C1), grey (C2), blue (C3), and dark red (C4) lines represent the detection at 254 nm for standard ellagic acid (at 40 μg/mL), MA (at 50 mg/mL), MACT (at 40 mg/mL), and CT (at 50 mg/mL), respectively. Finally, the red line (D1) for standard rutin, QUE, and KAF (at 40 μg/mL) detected at 370 nm (D1) aligns with the grey (D2), blue (D3), and dark red (D4) lines of MA (at 50 mg/mL), MACT (at 40 mg/mL), and CT (at 50 mg/mL) for quantification.

S2 Table

HPLC analysis of the phenolic/flavonoid contents in the MACT, MA, and CT. Data are presented as mean ± SD (carried out in triplicate).

| **Chemicals** | **Plant extracts (mg/g)** | | |
| --- | --- | --- | --- |
|  | **MA** | **CT** | **MACT** |
| Gallic acid | - | 0.231 ± 0.008 | 0.093 ± 0.005 |
| Rutin | 0.072 ± 0.001 | 0.754 ± 0.020 | 0.266 ± 0.005 |
| Quercetin | - | 0.021 ± 0.001 | 0.011 ± 0.000 |
| Kaempferol | - | 0.032 ± 0.000 | 0.037 ± 0.000 |
| Chlorogenic acid | 0.091 ± 0.001 | - | 0.064 ± 0.002 |
| p-Coumaric acid | - | - | - |
| Ellagic acid | 0.017 ± 0.000 | 0.224 ± 0.003 | 0.086 ± 0.001 |

- ; Not detected


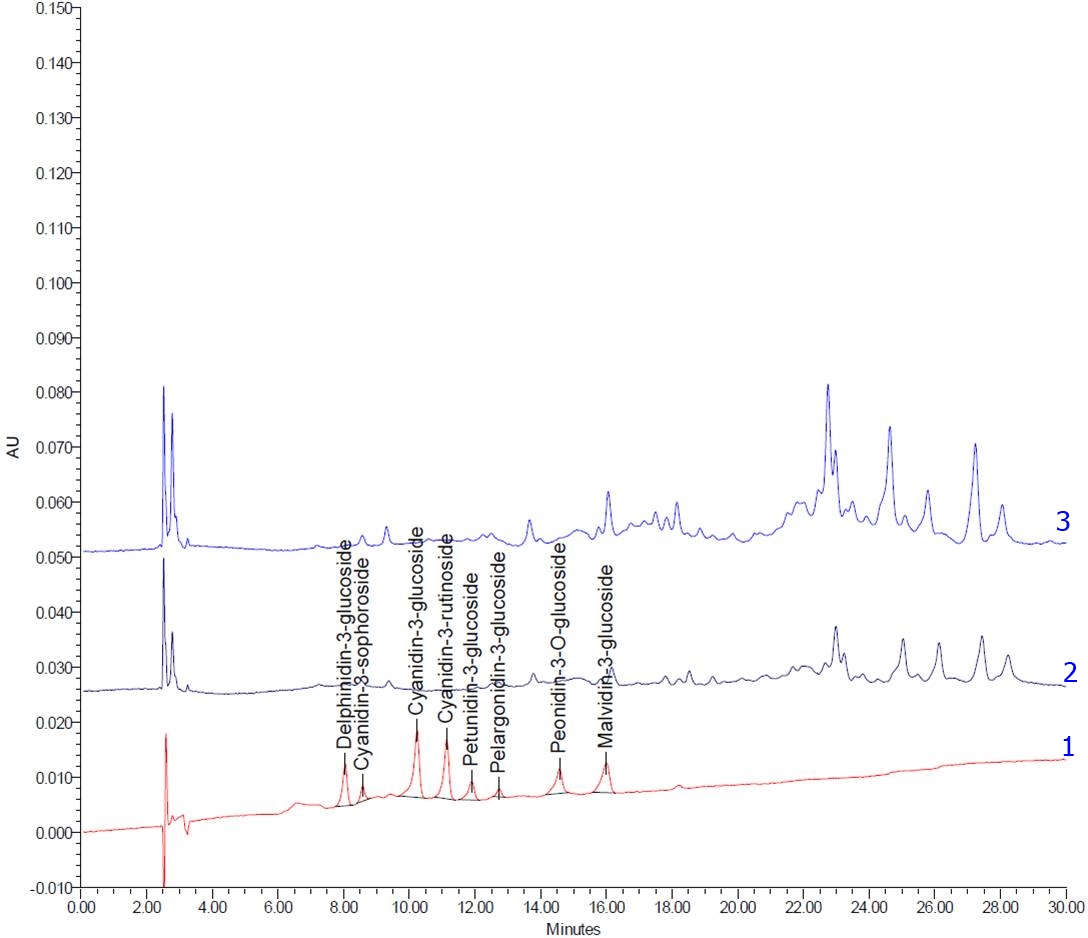


**(1)**

**(2)**

**(3)**

**Figure S3**

Chromatogram of plant extracts. Red line (1) was the standards of the anthocyanin including delphinidin-3-glucoside (Del3G), cyanidin-3-sophoroside (C3S), cyanidin-3-glucoside (C3G), cyanidin-3-rutinoside (C3R), petunidin-3-O-beta-D-glucoside (Pet3G), pelargonidin-3-glucoside (Pel3G), peonidin-3-O-glucoside (Peo3G), and malvidin-3-glucoside (M3G), respectively (at 5 μg/mL). The grey (2) and blue (3) lines were the MACT (at 40 mg/mL), and CT (at 20 mg/mL) were detected at 530 nm.

**S3 Table**

HPLC analysis of the anthocyanin contents in the MACT, MA, and CT. Data are presented as mean ± SD (carried out in triplicate).

| **Chemicals** | **Plant extracts (mg/g)** | |
| --- | --- | --- |
|  | **CT** | **MACT** |
| Delphinidin-3-glucoside | - | - |
| Cyanidin-3-sophoroside | 0.263 ± 0.013 | 0.332 ± 0.014 |
| Cyanidin-3-glucoside | - | - |
| Cyanidin-3-rutinoside | - | - |
| Petunidin-3-O-glucoside | - | - |
| Pelargonidin-3-glucoside | - | - |
| Peonidin-3-O-glucoside | - | - |
| Malvidin-3-glucoside | 0.222 ± 0.007 | 0.211 ± 0.019 |

- ; Not detected

**S4 Table**

Significant parameters of sedative-hypnotic, cognitive, and nootropic effects of MACT in a dose-dependent fashion and in comparison, to either MA or CT alone. Data are presented as mean ± S.E.M. (n=6/group)

| **Group of Treatments** | **Sleep latency (minutes)** | | **Sleep stage duration (minutes)** | | | **Number of bouts (times)** | **NOPI** | **Time spent in open arms (%)** | **Entries in the open arms (times)** |
| --- | --- | --- | --- | --- | --- | --- | --- | --- | --- |
|  | **NREM** | **REM** | **WAKE** | **NREM** | **REM** | **REM** |  |  |  |
| Control | 31.49 ± 3.66^c^ | 116.74 ± 12.61^d^ | 92.17 ± 5.00^d^ | 84.19 ± 5.14^a^ | 3.63 ± 0.61^a^ | 2.83 ± 0.40^a^ | -2.27 ± 4.04^a^ | 36.87 ± 3.19^a^ | 1.17 ± 0.38^a^ |
| MACT125 | 19.33 ± 1.35^b^ | 110.79 ± 10.39^cd^ | 85.39 ± 3.71^cd^ | 86.75 ± 3.42^ab^ | 7.86 ± 1.89^ab^ | 5.17 ± 0.87^a^ | 5.58 ± 4.39^ab^ | 40.88 ± 2.40^ab^ | 2.33 ± 1.25^ab^ |
| MACT250 | 15.74 ± 2.66^ab^ | 68.72 ± 3.19^ab^ | 64.67 ± 3.70^ab^ | 101.46 ± 2.67^bc^ | 13.88 ± 1.72^bc^ | 9.17 ± 1.05^bc^ | 12.76 ± 3.53^ab^ | 47.78 ± 3.15^abc^ | 3.67 ± 2.40^b^ |
| MACT500 | 7.04 ± 0.34^a^ | 46.46 ± 3.93^a^ | 50.63 ± 1.61^a^ | 110.51 ± 1.66^c^ | 16.86 ± 2.10^c^ | 10.67 ± 0.84^c^ | 31.11 ± 3.57^c^ | 59.10 ± 4.05^c^ | 5.67 ± 4.40^c^ |
| MA500 | 17.32 ± 2.52^ab^ | 83.71 ± 5.75^bcd^ | 68.79 ± 4.21^bc^ | 98.60 ± 4.99^abc^ | 12.61 ± 1.34^bc^ | 10.83 ± 0.87^c^ | 19.95 ± 2.77^bc^ | 52.63 ± 3.11^bc^ | 4.00 ± 3.06^bc^ |
| CT500 | 18.24 ± 3.14^b^ | 78.29 ± 8.54^abc^ | 72.40 ± 4.51^bc^ | 99.25 ± 3.79^abc^ | 8.35 ± 1.82^ab^ | 6.33 ± 1.28^ab^ | 15.41 ± 3.15^b^ | 51.01 ± 2.99^bc^ | 3.50 ± 2.40^b^ |
| F-test, *p*-value | [F(5, 30) = 9.604, *P* < 0.0001] | [F(5, 30) = 10.434, *P* < 0.0001] | [F(5, 30) = 14.265, *P* < 0.0001] | [F(5, 30) = 7.376, *P* < 0.0001] | [F(5, 30) = 8.420, *P* < 0.0001] | [F(5, 30) = 12.273, *P* < 0.0001] | [F(5, 30) = 10.219, *P* < 0.0001] | [F(5, 30) = 6.465, *P* < 0.0001] | [F(5, 30) = 13.031, *P* < 0.0001] |

The statistical differences were adjusted with a one-way ANOVA followed by Tukey's post hoc correction when the *p*-value was less than 0.05. A difference in symbolic letters means statistical differences within parameters.

**S5 Table**

Significant parameters of sedative-hypnotic, cognitive and anxiolytic effects of MACT in GABA_A_ receptor-dependent conditions. Data are presented as mean ± S.E.M. (n=6/group)

| **Group of Treatments** | **Sleep latency (minutes)** | | **Sleep stage duration (minutes)** | | | **Number of bouts (times)** | **NOPI** | **Time spent in open arms (%)** | **Entries in the open arms (times)** |
| --- | --- | --- | --- | --- | --- | --- | --- | --- | --- |
|  | **NREM** | **REM** | **WAKE** | **NREM** | **REM** | **REM** |  |  |  |
| Control | 31.49 ± 3.66^c^ | 116.74 ± 12.61^b^ | 92.17 ± 5.00^b^ | 84.19 ± 5.14^a^ | 3.63 ± 0.61^a^ | 2.83 ± 0.40^a^ | -2.27 ± 4.04^ab^ | 36.87 ± 3.19^ab^ | 1.17 ± 0.38^a^ |
| MACT500 | 7.04 ± 0.34^a^ | 46.46 ± 3.93^a^ | 50.63 ± 1.61^a^ | 110.51 ± 1.66^b^ | 16.86 ± 2.10^c^ | 10.67 ± 0.84^c^ | 31.11 ± 3.57^c^ | 59.10 ± 4.05^c^ | 5.67 ± 4.40^c^ |
| Bi | 27.71 ± 4.69^bc^ | 94.24 ± 7.13^b^ | 80.19 ± 7.54^b^ | 88.08 ± 7.15^a^ | 11.72 ± 1.64^bc^ | 8.17 ± 1.01^bc^ | -5.14 ± 2.41^a^ | 29.25 ± 2.58^a^ | 2.17 ± 0.48^a^ |
| BiMACT500 | 17.92 ± 2.79^ab^ | 96.07 ± 12.55^b^ | 74.63 ± 5.55^b^ | 97.57 ± 5.47^ab^ | 7.81 ± 1.15^ab^ | 7.00 ± 1.06^b^ | 10.40 ± 3.83^b^ | 44.44 ± 4.19^b^ | 4.00 ± 0.26^b^ |
| F-test, *p*-value | [F(3, 20) = 11.054, *P* < 0.0001] | [F(3, 20) = 9.248, *P* < 0.0001] | [F(3, 20) = 10.589, *P* < 0.0001] | [F(3, 20) = 5.767, *P* = 0.005] | [F(3, 20) = 14.460, *P* < 0.0001] | [F(3, 20) = 14.090, *P* < 0.0001] | [F(3, 20) = 21.981, *P* < 0.0001] | [F(3, 20) = 12.758, *P* < 0.0001] | [F(3, 20) = 25.088, *P* < 0.0001] |

The statistical differences were adjusted with a one-way ANOVA followed by Tukey's post hoc correction when the *p*-value was less than 0.05. A difference in symbolic letters means statistical differences within parameters.

**References**

[1] R. Vallat, M.P. Walker, An open-source, high-performance tool for automated sleep staging, Elife 10 (2021) 1–24. https://doi.org/10.7554/ELIFE.70092.

[2] J. Nukitram, D. Cheaha, N. Sengnon, J. Wungsintaweekul, S. Limsuwanchote, E. Kumarnsit, Ameliorative effects of alkaloid extract from Mitragyna speciosa (Korth.) Havil. leaves on methamphetamine conditioned place preference in mice, J Ethnopharmacol 284 (2022) 114824. https://doi.org/10.1016/j.jep.2021.114824.

[3] Z. Barger, C.G. Frye, D. Liu, Y. Dan, K.E. Bouchard, Robust, automated sleep scoring by a compact neural network with distributional shift correction, PLoS One 14 (2019) 1–18. https://doi.org/10.1371/journal.pone.0224642.

[4] M.J. Prerau, R.E. Brown, M.T. Bianchi, J.M. Ellenbogen, P.L. Purdon, Sleep neurophysiological dynamics through the lens of multitaper spectral analysis, Physiology 32 (2017) 60–92. https://doi.org/10.1152/physiol.00062.2015.
